# Outcomes of a Delirium Prevention Program in Older Persons After Elective Surgery: A Stepped-Wedge Cluster Randomized Clinical Trial

**DOI:** 10.1001/jamasurg.2021.6370

**Published:** 2021-12-15

**Authors:** Friederike Deeken, Alba Sánchez, Michael A. Rapp, Michael Denkinger, Simone Brefka, Juliane Spank, Carola Bruns, Christine A. F. von Arnim, Olivia C. Küster, Lars O. Conzelmann, Brigitte R. Metz, Christoph Maurer, Yoanna Skrobik, Oksana Forkavets, Gerhard W. Eschweiler, Christine Thomas

**Affiliations:** 1 Department of Social and Preventive Medicine, University of Potsdam, Potsdam, Germany; 2 Social and Preventive Medicine, Department of Sports and Health Sciences, Intrafaculty Unit of Cognitive Sciences, Faculty of Human Science, and Faculty of Health Sciences Brandenburg, Research Area Services Research and e-Health, University of Potsdam, Potsdam, Germany; 3 Agaplesion Bethesda Clinic Ulm, Institute for Geriatric Research, Ulm University, Geriatric Center Ulm, Ulm, Germany; 4 Department of Cardiothoracic and Vascular Surgery, Ulm University Hospital, Ulm, Germany; 5 Department of Geriatric Psychiatry and Psychotherapy, Klinikum Stuttgart, Stuttgart, Germany; 6 Division of Geriatrics, University Medical Center Goettingen, Göttingen, Germany; 7 Department of Neurology, University Hospital Ulm, Ulm, Germany; 8 Helios Clinic for Cardiac Surgery Karlsruhe, Karlsruhe, Germany; 9 Geriatric Center Karlsruhe, ViDia Christian Clinics Karlsruhe, Karlsruhe, Germany; 10 Center for Geriatrics and Gerontology, Medical Center University of Freiburg, Freiburg, Germany; 11 Department of Medicine, McGill University, Montreal, Quebec, Canada; 12 Geriatric Center at the University Hospital Tübingen, Tübingen, Germany; 13 Department of Psychiatry and Psychotherapy, University Hospital of Tübingen, Tübingen, Germany

## Abstract

**Importance:**

Delirium significantly worsens elective surgery outcomes and costs. Delirium risk is highest in elderly populations, whose surgical health care resource consumption (50%) exceeds their demographic proportion (15% to 18%) in high-resource countries. Effective nonpharmacologic delirium prevention could safely improve care in these vulnerable patients, but data from procedure-specific studies are insufficiently compelling to drive changes in practice. Delirium prevention approaches applicable to different surgical settings remain unexplored.

**Objective:**

To examine whether a multifaceted prevention intervention is effective in reducing postoperative delirium incidence and prevalence after various major surgical procedures.

**Design, Setting, and Participants:**

This stepped-wedge cluster randomized trial recruited 1470 patients 70 years and older undergoing elective orthopedic, general, or cardiac surgery from November 2017 to April 2019 from 5 German tertiary medical centers. Data were analyzed from December 2019 to July 2021.

**Interventions:**

First, structured delirium education was provided to clinical caregivers at each site. Then, the study delirium prevention team assessed patient delirium risk factors and symptoms daily. Prevention was tailored to individual patient needs and could include: cognitive, motor, and sensory stimulation; meal companionship; accompaniment during diagnostic procedures; stress relaxation; and sleep promotion.

**Main Outcomes and Measures:**

Postoperative delirium incidence and duration.

**Results:**

Of 1470 included patients, 763 (51.9%) were male, and the median (IQR) age was 77 (74-81) years. Overall, the intervention reduced postoperative delirium incidence (odds ratio, 0.87; 95% CI, 0.77-0.98; *P* = .02) and percentage of days with delirium (intervention, 5.3%; control, 6.9%; *P* = .03). The effect was significant in patients undergoing orthopedic or abdominal surgery (odds ratio, 0.59; 95% CI, 0.35-0.99; *P* = .047) but not cardiac surgery (odds ratio, 1.18; 95% CI, 0.70-1.99; *P* = .54).

**Conclusions and Relevance:**

This multifaceted multidisciplinary prevention intervention reduced postoperative delirium occurrence and days with delirium in older patients undergoing different elective surgical procedures but not cardiac procedures. These results suggest implementing this delirium prevention program will improve care and outcomes in older patients undergoing elective general and orthopedic procedures.

## Key Points

**Question:**

Does a multimodal nonpharmacological approach prevent delirium in older patients undergoing elective surgical procedures?

**Findings:**

This stepped-wedge cluster trial recruited 1470 patients 70 years and older who were randomized in 5 clusters to patient-centered evidence-based intervention (ie, personalized stimulation, company, relaxation) vs routine care. The intervention reduced delirium incidence after various major procedures, most significantly in patients undergoing noncardiac surgery; the intervention did not change cardiac surgery postoperative delirium incidence.

**Meaning:**

Results of this stepped-wedge cluster trial suggest the implementation of this multimodal nonpharmacological delirium prevention program may improve delivery of targeted care and patient outcomes in older patients undergoing elective noncardiac surgical procedures.

## Teaser

This stepped-wedge cluster randomized clinical trial examines whether a multifaceted prevention intervention is effective in reducing postoperative delirium incidence and prevalence among older patients after various major surgical procedures.

## Introduction

Postoperative delirium is frequent in older patients. Its association with higher mortality,^1,2^ cognitive decline,^3^ loss of autonomy, and increased hospitalization and costs^4,5^ warrant preventive initiatives in frail or high-risk patients.^4,5,6^ The 16% of US individuals 65 years and older accounted for more than 40% of surgical interventions in 2019.^7^ This demographic/surgical health care utilization disproportion will worsen as the number of people 65 years and older is expected to double by 2050. In medical patients, one-third of delirium cases are considered preventable^8,9,10^ with multimodal nonpharmacological interventions.^10,11,12^ Once delirium occurs, no treatment changes its course or outcome,^13^ highlighting the importance of postoperative delirium prevention.

Patient frailty is related to age older than 65 years^14^ and best predicts delirium risk.^15,16^ Postoperative delirium rates vary (11% to 65%^15,17,18^); studies addressing risk,^12,19,20,21,22^ precipitating factors, and prevalence focus on single types of surgery.^23^ Hip fractures, an emergency procedure, represent most of the intervention literature. To our knowledge, no study stratifies for delirium risk or includes frail patients and/or patients with dementia. Large-scale studies incorporating different elective surgical interventions have not been published.^11,15,23,24^

We compared the efficacy of a multimodal, multidisciplinary, nonpharmacological intervention in patients 70 years and older undergoing various elective major surgical procedures. We hypothesized our multimodal individualized best practice–based intervention would reduce postoperative delirium incidence (our primary outcome) and shorten delirium duration.

## Methods

### Trial Oversight

The Patientensicherheit, Wirtschaftlichkeit und Lebensqualität (PAWEL; ie, patient safety, cost-effectiveness, and quality of life) study randomized 3 German university hospitals (Tübingen, Freiburg, and Ulm), and 2 German tertiary care (Stuttgart and Karlsruhe) center clusters. The first of the stepped-wedge model’s seven 12-week periods had no intervention. Then, every 12 weeks, 1 cluster was randomized to the prevention protocol. All 5 clusters thus implemented the intervention for at least 12 weeks by the end of the study. The Tübingen Faculty of Medicine’s Ethics Commission, Potsdam and Ulm Universities, and the District Physicians Chamber of Baden-Württemberg provided ethical approval. All patients or substitute decision-makers provided written informed consent. The trial protocol can be found in Supplement 1. This study followed the Consolidated Standards of Reporting Trials Extension (CONSORT Extension) reporting guideline for stepped-wedge cluster randomized trials.

Center eligibility was based on willingness and at least 900 major procedures per year in older adults. To balance procedures within and across centers, recruitment was capped in individual sites and in study intervals at 66% for: cardiac/vascular; orthopedic; and (combined) intra-abdominal, urological, or thoracic surgery procedures. Patients were assessed preoperatively, then daily up postoperative day 7 and on discharge.^25^

### Participants

Patients 70 years and older undergoing major elective surgery with an expected cut-to-suture-time of 60 minutes or more were eligible. Recruitment was conducted by a site-based independent medical specialist. Patients requiring emergency surgery, patients who did not provide consent, and and patients with an expected survival of 15 months or less were excluded.^25^

### Randomization

Cluster randomization sequences (5 clusters) were computer generated. Training teams coached local trainers and then clinical staff during 6 weeks prior to the center’s intervention implementation. Allocation was concealed both at the cluster level and the patient level; only the consortium leader and staff generating random sequences knew the randomization allocation. Participants and outcome assessors remained blinded.

### Procedures

The intervention (AKTIVER [“More Active”]: Alltags- und Kognitions-Training & Interdisziplinarität verbessert Ergebnis und mindert das Risiko [“everyday skills and cognition training and interdisciplinarity improves outcome and mitigates risk”]) included 7 best-practice delirium prevention modules: cognitive, motor, and sensory stimulation; meal companionship; diagnostic test and operation room accompaniment; stress relaxation; and sleep promotion. Patient needs and preferences determined module deployment. Our AKTIVER manual provided explicit parameters for each module. The authors (J.S., C.B., and C.T.) constructed AKTIVER using evidence (Care of Confused Hospitalized Older Persons,^26^ Hospital Elderly Life Program,^27^ and others^10^), inspiration from other programs,^28,29,30,31,32,33^ and their clinical experience^25^ (Table 1).^34,35,36,37,38,39,40,41,42,43^

**Table 1.** AKTIVER Delirium Prevention Program

Section of AKTIVER program	Content	Population trained and delirium risk factors addressed	Main indications	Model
**Staff education^a^**				
Basic education (1.5 h)	Delirium primer, symptoms, outcome, prevention options, management	Nurses, physicians, aides, therapists, OR personnel, cleaning and transportation staff	Delirium symptom recognition, delirium awareness	CHOPs,^b^ principle 6
Delirium prevention level (10 h)	Delirium risk, adequate communication with patients with cognitive impairment	Nurses, therapists, physicians	Delirium risk and prevention, communication with patients with cognitive impairment	CHOPs, principle 6; POD^c^
Delirium advocacy level (30 h in addition to delirium prevention level)	Delirium screening, risk factors and modifiable risk, detection of depression and dementias, pain assessment (especially in patients with cognitive impairment), detection and management of psychotic symptoms, sleep-wake rhythm, hyperactive states and apathy	Nurses, therapists, physicians	Delirium management, risk evaluation, prevention	CHOPs, principle 6; POD
**Tasks of delirium expert nurse**				
Risk assessment (preadmission and daily)	Checking present unmodifiable and modifiable risk factors, minimizing polypharmacy and inappropriate medication (beginning and daily updates)	Age^4,5,6,15,16,18,21,22,34^ (SIGN evidence level, IIIb-IIb), comorbidity^4,5,6,10,15,18,34^ (SIGN evidence level, IIb-Ic), depression^15,21^ (SIGN evidence level, IIIb-IIb), polypharmacy^4,10,15,34^ (SIGN evidence level, IIb), infection^4,16,18^ (SIGN evidence level, IIb), electrolyte disturbance^4,15,20^ (SIGN evidence level, IIb), frailty^4,10,12,15,16,18,34^ (SIGN evidence level, IIa), sensory deficits^4,5,6,10,12,15,16,18,34^ vision and hearing (SIGN evidence level, Ia-IIb), cognitive dysfunction^4,5,10,12,15,16,18,19,20,34^ (SIGN evidence level, IIb-Ic), sleep^15,16,22^ (SIGN evidence level, IIa), delirium history^16,18^	Planning of individual prevention schedule and frequency of modules	CHOPs, principles 1, 2, 3, and 5; DemDel^d^; HELP^e^; help+^f^
Rounds and patient visits (daily)	Patient assessment (pain, attention, orientation, psychomotor activity, psychotic symptoms), consultation with the interprofessional team on unit and family members, mobilization barriers (daily)	Pain^4,5,10,12,15,16,18,34^ (SIGN evidence level, Ia-IIb), cognition/cognitive dysfunction^4,5,10,12,15,16,18,19,20,34^ (SIGN evidence level, IIb-Ic), stress^15,16,20,34^ (SIGN evidence level, IV), medication^4,10,15,18,19,34^ (SIGN evidence level, Ib-IIa), sleep-wake cycle^15,16,22^ (SIGN evidence level, IIa), immobilization^16,18,19^ (SIGN evidence level, Ia-IIb), polypharmacy^4,5,6,18,34^ (SIGN evidence level, Ia-IIb)	Assessment of delirium symptoms, pain, new medication, training of case-based risk management, delirium awareness for interprofessional team^36,37^	CHOPs, principles 1 to 7; DemDel; help+
Module assignment (daily)	Collecting information from patient, family, and ward team, including preferences and aversions (Sunflower Tool); modules can be adapted during the hospital stay (beginning and daily updates)	See below	Individualization of delirium prevention according to risks	CHOPs, principles 4, 5, and 7; DemDel; HELP; help+
Team handover (daily)	Discuss symptoms and daily well-being of patients, check their individual needs (pain, fluids, stress reduction) to optimize prevention, involving family if possible; microteaching and supervision (twice daily)	Pain^4,5,10,12,15,16,18,34^ (SIGN evidence level, Ia-IIb), stress^16,18,19,34,38^ (SIGN evidence level, IV), disorientation^4,5,10,12,16,18,34^ (SIGN evidence level, IIb-Ic), dehydration^4,15,18^ (SIGN evidence level, Ia-IIb), anxiety,^15,21^ apathy^15^	Information sharing, module implementation planning, shift plans, teaching	DemDel; HELP; help+
**Intervention modules** ^g^				
Orientation visit	Naming the daily schedule, clock, calendar, bathroom sign, verbal orientation, put clean glasses on or insert hearing aids	Vision impairment^4,5,6,18,34^ (SIGN evidence level, Ic), hearing impairment^4,5,6,18,34^ (SIGN evidence level, Ia-IIb), cognitive dysfunction^4,5,6,10,12,15,16,18,19,20,34^ (SIGN evidence level, IIb-Ic), stress^15,16,18,19,34^ (SIGN evidence level, IV), anxiety^15,21^	Reorientation, stress reduction	CHOPs, principles 4, 5, and 7; HELP; help+; mHELP^h^; POD
Mobilization	Motivation and activation to simple movement exercises, accompaniment during mobilization in bed or for walks	Immobilization^16,18,19^ (SIGN evidence level, Ia-IIb), frailty^4,10,12,15,16,18,34^ (SIGN evidence level, IIa)	Mobilization	DemDel; HELP; mHELP
Activation visit	Cognition promotion (eg, games, Sudoku, quiz, reading newspaper)	Cognitive dysfunction^4,5,10,12,15,16,18,19,20,34^ (SIGN evidence level, IIb-Ic)	Cognitive activation	CHOPs, principles 4, 5, and 7; HELP; help+
Meal accompaniment	Company during meals, support with meal arrangement, fluid intake	Malnutrition^15^ (SIGN evidence level, IIb), dehydration^6,10,12,16,18,34^ (SIGN evidence level, IIb)	Nutrition, fluid intake	HELP; help+; mHELP; POD
Relaxation/sleep promotion	Music, warm drinks, acupressure, relaxation exercises	Anxiety,^15,21^ sleep promotion^15,16,22^ (SIGN evidence level, IIa), stress^15,16,18,19,34^ (SIGN evidence level, IV)	Stress reduction, sleep-wake rhythm	HELP; help+
Diagnostic chaperonage	Accompanying patients to examinations (eg, CT, ECG), activation, orientation	Anxiety,^15,21^ stress^15,16,18,19,34^ (SIGN evidence level, IV), change of environment/transfer^16,18^	Stress reduction, orientation	The Elderly in the OR^i^
Attendance to the OR	Accompanying to the OR sluice, first contact in the recovery room, reorientation, providing sensory aids	Anxiety,^15,21^ stress^15,16,18,19,34^ (SIGN evidence level, IV), change of environment/transfer^16,18^ (SIGN evidence level, IV), pain^4,5,10,12,15,16,18,19,34^ (SIGN evidence level, Ia-IIb), vision and hearing impairment^4,5,6,10,12,15,16,18,34^ (SIGN evidence level, Ia-IIb)	Provide familiarity, reorientation, stress reduction	The Elderly in the OR

Abbreviations: CHOP, Care of Confused Hospitalised Older Persons; CT, computed tomography; DemDel, Basel Dementia and Delirium Prevention and Management Program; ECG, electrocardiography; HELP, Hospital Elder Life Program; help+, adapted Hospital Elder Life Program plus consultation/liaison physician; mHELP, modified Hospital Elder Life Program; OR, operating room; POD, Prevention of Delirium; SIGN, Scottish Intercollegiate Guidelines Network.

^a^ Tabet et al^39^ evaluated the efficacy of staff education to prevent delirium in a medical ward. The incidence of delirium was significantly lower on the intervention ward despite a wide confidence interval (odds ratio, 0.45; 95% CI, 0.26-0.96). Among single-component interventions, only staff education, reorientation protocol (GRADE evidence: very low), and Geriatric Risk Assessment MedGuide software (hazard ratio, 0.42; 95% CI, 0.35-0.52; GRADE evidence: moderate) were effective in preventing delirium.^24^

^b^ Implementation of a model of care for hospitalized older persons with cognitive impairment (the CHOPs) in 6 New South Wales hospitals.^26^ Settings included hospital-wide departments, surgery departments, medicine departments, intensive care units, and emergency departments in 22 hospitals in New South Wales, Australia, guided by 7 key principles.^40^

^c^ Settings for the POD program included medical and surgical departments in 8 National Health Service hospitals in Great Britain.^28,29^

^d^ The DemDel program is a before-and-after study of a nurse-led comprehensive delirium management program for older acute care inpatients with cognitive impairment.^33^ The program also used data from the interdisciplinary nurse-led Delirium Prevention and Management Program on nursing workload.^32^ Settings included hospital-wide rollouts, surgery departments, medicine departments, and emergency departments in a university hospital and a tertiary care hospital in Basel, Switzerland.

^e^ Settings for the HELP program included predominantly medical (and geriatric) departments, some surgical departments, few hospital-wide rollouts, emergency departments, and rehabilitation facilities in more than 80 hospitals in the US, Canada, and Australia and in single sites in Europe.^27,31,41^

^f^ The help+ program has adapted the HELP program to the German health care system and included a psychogeriatric consultation/liaison physician.^30,42^ Settings included medical, surgical, and neurological departments in a tertiary care university hospital in Bielefeld, Germany.

^h^ The setting for the mHELP program was an abdominal surgery department in a tertiary care hospital in Taipeh, Taiwan.^37^

^i^ Settings for The Elderly in the OR program included surgical departments, ORs, and intensive care units in a tertiary care hospital in Münster, Germany, and in several other hospitals in Germany.^43^

At each site, more than 70% of hospital staff completed a 90-minute basic lesson in delirium detection, management, and prevention; 20% completed additional 10-hour and 10% completed 30-hour delirium advocacy courses.^24,35,39,44^ Local psychogeriatric nurse specialists received 80 hours of training in delirium risk detection, assessments, prevention, medication surveillance, daily evaluations, and prescribing individual prevention modules. After 40 hours of content/implementation training for 7 intervention modules, the independent delirium study prevention team observed patients throughout hospitalization and provided the intervention modules several times a day as needed. This team consisted primarily of 2 psychogeriatric nurses working 20 hours per week, supported by 3 to 5 volunteer aides (about 100 hours per week). Compliance and reliability across sites were ensured by one team training each center’s staff, a reference manual, unannounced on-site visits, and documentation reviews.

### Outcomes

Trained clinicians assessed all outcomes. Blinding was ensured by suggesting these data would serve for delirium risk score validation.

Delirium, the primary outcome, was assessed daily with the validated Confusion Assessment Method (CAM),^45^ I-Confusion Assessment Method (I-CAM)^46^ between 1 and 6 pm (7 postoperative days), followed by a validated postdischarge medical record review. Delirium symptoms fluctuate^47^; medical record reviews capture findings absent during CAM assessments, as described by others.^48^ Individual study participant data, and not clusters, determined outcome measurements.

The German I-CAM^46^ was operationalized further; we assessed structured attention and logic and identified abnormal psychomotor activity to classify delirium subtype. These modifications harmonized CAM screening with *International Statistical Classification of Diseases and Related Health Problems, Tenth Revision *delirium diagnostic criteria. Delirium duration was assessed by medical record review. We tallied days with delirium in all patients, mean delirium days, and percentage of days with delirium in each study group.

Baseline data included cognitive function (Montreal Cognitive Assessment [MoCA]),^49^ subjective memory impairment (SMI),^50^ comorbidities (Charlson Comorbidity Index [CCI]),^51^ visual impairment,^52^ depression (4-item Patient Health Questionnaire [PHQ-4]),^53^ functional status,^54^ and frailty (Canadian Study of Health and Aging [CSHA] Clinical Frailty Scale [CFS]).^55^ Polypharmacy meant routine administration of 5 or more daily medications.^56^ Anesthesia duration was the time from induction until extubation.

We recorded adverse events, including falls, strokes, infections, and significant perioperative complications (death, reoperation, pneumonia, sepsis). An external Regional Ethics and Data Monitory Board assessed feasibility and serious adverse event occurrence every 3 months and performed yearly audits.

### Statistical Analysis

The power analysis for the primary outcome, delirium incidence, assumed a 25% to 15%^11^ reduction after intervention. A Fisher exact test conventional analysis detected delirium proportion differences, given a power of 1 − β = 0.80 and an α error of 5%, showed that the study would require 514 patients with a 1:1 randomization. Woertmann adjustment^57^ for 5 crossing points in the stepped-wedge design, with 50 patients maximum per cluster per period and a 0.01 intracluster correlation, led to a correction factor of 2.63; this lead to a target population of 514 × 2.63 = 1351 patients. Assuming a 15% dropout rate increased the estimated sample to 1500 patients (750 per arm).^25^

Sample characteristics were summarized as frequencies and percentages for categorical variables and medians and IQRs for nonparametric continuous variables. Normality was established using Shapiro-Wilk tests. Baseline intervention and control group differences in bivariate analyses were examined using Mann-Whitney *U* tests for nonparametric continuous variables (age, education [years], Barthel Index score, MoCA score, CCI score, body mass index, CSHA-CFS score), multinomial regression for categorical variables (marital status, type of surgery), and χ^2^ tests for binary variables (sex, subjective memory impairment, visual impairment, dementia [CCI score], depression (PHQ-4 score), polypharmacy, sleeping medication use, current daily smoker, current alcohol misuse). We calculated risk ratios (RRs) for binary variables, relative RRs (RRRs) for categorical variables, and median differences for continuous variables. Median differences with 95% CIs of the difference were estimated using the Hodges-Lehman estimator. Significantly different variables (intervention vs control groups) were included as covariates in the generalized estimating equation (GEE) models. Odds ratios (ORs), RRs, and RRRs for delirium were calculated in intervention and control groups. An intraclass correlation coefficient was obtained for all clusters. Interrater reliability (IRR) was established for I-CAM assessments and medical record reviews across raters and centers.^58^

Primary outcome data were available for all participants, reducing the 1500 patient requirement to 1351. For GEE model covariates, missing data occurred in less than 5%, accounting for 0% to 3.7% (eTable 3 in Supplement 2). The Little test of missing completely at random was not significant (χ^2^_23_ = 31.56; *P* = .11). For secondary analysis variables, data were missing in less than 0.5%. The GEE model examined the intervention’s effect on delirium, using the center as the subject variable. We assumed a binomial distribution, a logit link, and an exchangeable correlation structure.

For the first model, all variables with significantly different baselines (intervention vs control groups) were considered explanatory covariates (model 1). Four established major risk factors (age, MoCA score, dementia, and polypharmacy) were added in the second model (model 2). In model 3, we stratified the second model for cardiac and vascular surgery (hereafter referred to as cardiac surgery) (model 3a) vs noncardiac surgery (model 3b). The same GEE models with a Poisson distribution and log-link function were applied to obtain RRs and 95% CIs^58^ and to calculate RRRs.^59^

In secondary analyses, Mann-Whitney *U* testing assessed differences between intervention and control groups overall delirium days and percentages of days with delirium in the total sample and subgroups. We conducted the same GEE models in a subsample of patients with baseline frailty, ie, patients with CSHA-CFS^60^ scores of 5 or more.

Tests were 2-tailed, and statistical significance was set at a *P* value less than .05. Statistical analyses were performed using SPSS version 26 (IBM).

## Results

Between November 21, 2017, and April 12, 2019, 4113 patients were screened and 1470 recruited in the study (Figure). Of 1470 included patients, 763 (51.9%) were male, and the median (IQR) age was 77 (74-81) years.

**Figure.**
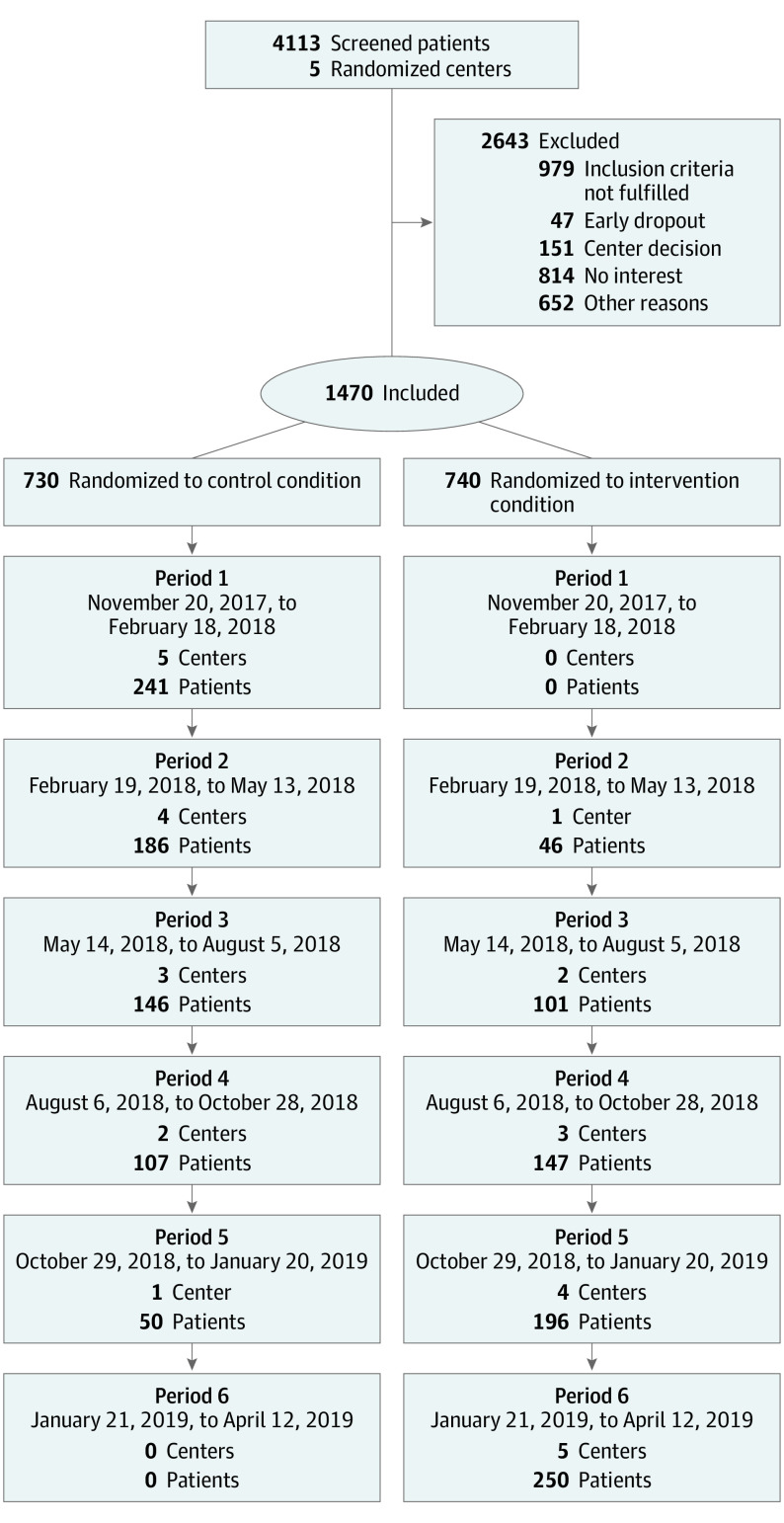
Recruitment CONSORT Flowchart

Group baseline demographic characteristics and surgical interventions appear in Table 2, and surgical procedures are shown in eTable 2 in Supplement 2. Age, marital status, visual impairment, and cognitive status (MoCA score) and functional status (Barthel Index score) were similar. While independent in everyday functioning, 1101 of 1470 patients (74.9%) presented at least mild baseline cognitive deficits. Compare with patients in the intervention group, those in the control group had fewer education years (12.5 vs 13.0; *P* < .001) and slightly higher frailty (mean CSHA-CFS score, 3.6 vs 3.4; *P* = .001; median difference, 0). The intervention group had more male patients (54.5% vs 49.3%; *P* = .048; RR, 1.10; 95% CI, 1.00-1.22), slightly more comorbidities (mean CCI score, 2.6 vs 2.1; *P* < .001; median difference, 0), and more subjective memory impairment (57.3% vs 49.2%; *P* = .005; RR, 1.15; 95% CI, 1.05-1.27). Patient populations differed by center before randomization (eTable 1 in Supplement 2); primary analyses were therefore conducted controlling for cluster effects.

**Table 2.** Baseline Characteristics of Participants

Characteristic	No./total No. (%)		*P* value	RR (95% CI)	RRR (95% CI)	Median difference (95% CI)
	Intervention group (n = 740)	Control group (n = 730)				
Age, median (IQR; range), y	78 (74 to 81; 70 to 98)	78 (74 to 81; 70 to 96)	.84	NA	NA	0 (−1.0 to 0)
Male	403/740 (54.5)	360/730 (49.3)	.048	1.10 (1.00 to 1.22)	NA	NA
Marital status						
Married, living together	466/739 (63.1)	448/730 (61.4)	.78	NA	1.07 (0.85 to 1.34)	NA
Married, living separately	45/739 (6.1)	48/730 (6.6)		NA	0.96 (0.62 to 1.50)	NA
Single, divorced, or widowed	228/739 (30.8)	234/730 (32.0)		NA	1 [Reference]	NA
Education, median (IQR), y	12 (12 to 15.5)	12 (10 to 13)	<.001	NA	NA	0
Barthel Index score, median (IQR)	100 (95 to 100)	100 (95 to 100)	.62	NA	NA	0
MoCA score, median (IQR)	24 (21 to 26)	24 (21 to 26)	.26	NA	NA	0 (−1.0 to 0)
SMI	422/736 (57.3)	359/729 (49.2)	.003	1.15 (1.05 to 1.27)	NA	NA
Visual impairment	327/677 (48.3)	320/696 (46.0)	.39	1.05 (0.94 to 1.18)	NA	NA
CCI score, median (IQR)	2 (1 to 4)	2 (1 to 3)	<.001	NA	NA	0 (0 to 1.0)
Dementia (by CCI questionnaire)	16/740 (2.2)	12/730 (1.6)	.47	1.32 (0.63 to 2.76)	NA	NA
Depression (PHQ-4 score >3)^53^	131/696 (17.7)	118/707 (16.2)	.30	1.13 (0.90 to 1.41)	NA	NA
BMI, median (IQR)^a^	26.5 (24.1 to 29.3)	26.6 (24.0 to 30.0)	.68	NA	NA	−0.9 (−0.5 to 0.4)
CSHA-CFS score, median (IQR)	3 (3 to 4)	3 (3 to 4)	.001	NA	NA	0
Polypharmacy^b^	453/707 (64.1)	481/709 (67.8)	.14	0.94 (0.88 to 1.02)	NA	NA
Sleeping medication use (last 4 wk)	108/683 (15.8)	138/706 (19.6)	.068	.81 (0.64 to 1.02)	NA	NA
Current daily smoker^c^	32/736 (4.3)	31/730 (4.2)	.14	0.98 (0.80 to 1.20)	NA	NA
Current alcohol misuse^d^	4/733 (0.5)	2/727 (0.3)	.42	1.98 (0.36 to 10.78)	NA	NA
Type of surgery						
Cardiac or vascular surgery	273/740 (36.9)	259/730 (35.5)	<.001	NA	0.34 (0.18 to 0.66)	NA
Orthopedic/spine surgery	352/740 (47.6)	390/730 (53.4)		NA	0.29 (0.15 to 0.56)	NA
Abdominal surgery	75/740 (10.1)	68/730 (9.3)		NA	0.36 (0.18 to 0.73)	NA
Other surgery	40/740 (5.4)	13/730 (1.8)		NA	1 [Reference]	NA
Length of anesthesia, median (IQR), min	183 (145 to 276)	185 (141 to 264)	.56	NA	NA	2.0 (−5.0 to 10.0)

Abbreviations: BMI, body mass index; CCI, Charlson Comorbidity Index; CSHA-CFS, Canadian Study of Health and Aging Clinical Frailty Scale; MoCA, Montreal Cognitive Assessment; NA, not applicable; PHQ-4, 4-item Patient Health Questionnaire; RR, risk ratio; RRR, relative risk ratio; SMI, subjective memory impairment.

^a^ Calculated as weight in kilograms divided by height in meters squared.

^b^ Polypharmacy defined as routine administration of 5 or more daily medications.

^c^ Defined as smoking 5 or more cigarettes per day.

^d^ Defined as the use of 3 or more drinks daily.

The intraclass correlation coefficient across centers revealed a value of −0.58, indicating random distribution. Comparing 86 I-CAM scores and 20 medical record reviews, the I-CAM score’s IRR (Krippendorff α = 0.73; 95% CI, 0.48-0.94) was satisfactory and the medical record review’s IRR (Krippendorff α = 0.85; 95% CI, 0.79-0.90) was good. Site visit checklist evaluation showed high adherence to recommended prevention measures and intervention fidelity within individual centers.^61^

Delirium occurred in 318 patients (21.6%) in the total sample, 190 (35.7%) of those undergoing cardiac procedures, and 128 (13.6%) of those undergoing noncardiac procedures. Preventive intervention led to lower new delirium proportions overall (19.9% vs 23.4%; RR, 0.85; 95% CI, 0.70-1.03; *P* = .10; RRR, 15.2%; 95% CI, −3.1 to 30.2). Striking outcome differences between cohorts led to surgery type-based stratification. Delirium rates in patients undergoing noncardiac surgery (n = 938) were significantly lower in the intervention group compared with the control group (10.9% vs 16.3%; RR, 0.67; 95% CI, 0.48-0.93; *P* = .008; RRR, 33.2%; 95% CI, 7.1-52.0).

The intervention and control groups were no different in patients undergoing cardiac surgery (Table 3). Delirium occurrence by period and center is depicted in eTable 4 in Supplement 2.

**Table 3.** Delirium Occurrence

Outcome	No. (%)			Odds ratio (95% CI)	RR (95% CI)	*P* value	RRR (95% CI), %
	Intervention	Control	Total				
Total sample, No.	740	730	1470	NA	NA	NA	NA
Delirium	147 (19.9)	171 (23.4)	318 (21.6)	0.81 (0.63 to 1.04)	0.85 (0.70 to 1.03)	.10	15.2 (−3.1 to 30.2)
No delirium	593 (80.1)	559 (76.6)	1152 (78.4)				
Cardiac surgery, No.	273	259	532	NA	NA	NA	NA
Delirium	96 (35.2)	94 (36.5)	190 (35.7)	0.95 (0.67 to 1.36)	0.97 (0.77 to 1.22)	.79	3.1 (−21.7 to 22.9)
No delirium	177 (64.8)	165 (63.7)	342 (64.3)				
Noncardiac surgery, No.	467	471	938	NA	NA	NA	NA
Delirium	51 (10.9)	77 (16.3)	128 (13.6)	0.63 (0.43 to 0.92)	0.67 (0.48 to 0.93)	.008	33.2 (7.1 to 52.0)
No delirium	416 (89.1)	394 (83.7)	810 (86.4)				

Abbreviations: NA, not applicable; RR, risk ratio; RRR, relative risk ratio.

The effect of our multimodal intervention was demonstrated in the primary GEE analysis (model 1). Delirium is the dependent variable, and intervention the primary explanatory variable, while controlling for differing baseline characteristics in both the intervention and control groups and outcomes (Table 4). A significant inverse association between the intervention and delirium incidence was found (OR, 0.87; 95% CI, 0.77-0.98; *P* = .02), indicating the substantial delirium risk reduction predicted in the primary analysis.

**Table 4.** Generalized Estimating Equation Analysis (Model 1^a^) With Delirium as Dependent Variable^b^

Variable	Coefficient, β (SE)	Wald χ^2^	OR (95% CI)	*P* value	RRR (95% CI), %
Constant	−2.52 (0.73)	11.994	0.08 (0.02 to 0.33)	.001	91.6 (74.9 to 97.2)
Intervention (intervention group)	−0.14 (0.06)	5.380	0.87 (0.77 to 0.98)	.02	9.2 (−1.9 to 19.1)
Male	0.66 (0.14)	21.298	1.93 (1.46 to 2.55)	<.001	−61.2 (−106.7 to −25.8)
Education (years)	−0.05 (0.03)	2.65	0.95 (0.89 to 1.01)	.10	3.6 (−1.0 to 8.1)
SMI	0.13 (0.15)	0.784	1.14 (0.85 to 1.54)	.38	−9.7 (−35.8 to 11.3)
CCI score	0.01 (0.04)	0.104	1.01 (0.94 to 1.09)	.75	−0.8 (−5.9 to 4.1)
CSHA-CFS score	0.42 (0.06)	56.085	1.52 (1.36 to 1.69)	<.001	−34.4 (−45.6 to −24.1)
Type of surgery^c^					
Cardiac or vascular	0.77 (0.39)	3.970	2.16 (1.01 to 4.61)	.046	−77.3 (−192.7 to −7.4)
Orthopedic/spine	−0.71 (0.33)	4.571	0.49 (0.26 to 0.94)	.03	39.7 (5.7 to 61.5)
Abdominal	−0.36 (0.17)	4.608	0.70 (0.50 to 0.97)	.03	23.8 (1.6 to 40.9)

Abbreviations: CCI, Charlson Comorbidity Index; CSHA-CFS, Canadian Study of Health and Aging Clinical Frailty Scale; RRR, relative risk ratio; SMI, subjective memory impairment.

^a^ Adjusted for all variables that were significant between the intervention group (n = 718) and control group (n = 708) at baseline.

^b^ Quasi-likelihood under independence model criterion = 1368.165. Corrected quasi-likelihood under independence model criterion = 1334.618.

^c^ Reference category was other surgery.

Frailty (as measured by CSHA-CFS score; OR, 1.52; 95% CI, 1.36-1.69; *P* < .001) or male sex (OR, 1.93; 95% CI, 1.46-2.55; *P* < .001) were significantly associated with delirium. Undergoing cardiac surgery was associated with higher delirium incidence (OR, 2.16; 95% CI, 1.01-4.61; *P* = .046). The variance inflation factor value for the explanatory variables included in GEE model 1 ranged from 1.04 to 1.12, indicating the multicollinearity assumption was not violated.

The second GEE model (model 2) integrated 4 major delirium risk factors (eTable 5 in Supplement 2), and the effects were similar to model 1. Of the major risk factors, age (OR, 1.03; 95% CI, 1.01-1.06; *P* = .01) and dementia (OR, 4.44; 95% CI, 1.61-12.28; *P* = .004) were associated with delirium, while MoCA scores were inversely associated with delirium (OR, 0.89; 95% CI, 0.85-0.93; *P* < .001). The variance inflation factor value for the explanatory variables included in GEE model 2 ranged from 1.04 to 1.34.

Model 3 stratified for cardiac vs noncardiac surgery (eTable 6A and 6B in Supplement 2). The intervention and delirium were unrelated in patients undergoing cardiac surgery (OR, 1.18; 95% CI, 0.70-1.99; *P* = .54). For patients undergoing noncardiac surgery, the intervention was significantly inversely associated with delirium incidence (OR, 0.59; 95% CI, 0.35-0.99; *P* = .047). Adding cardiopulmonary bypass as a covariate to the cardiac surgery group did not change the outcomes (OR, 2.15; 95% CI, 0.99-4.62; *P* = .05).

To account for a time effect in the model, an interaction term between the intervention and duration in intervention variables was added to models 3a and 3b. Similar intervention effects were found in those undergoing noncardiac procedures (OR, 0.50; 95% CI, 0.27-0.95; *P* = .03) and cardiac surgery procedures (OR, 1.48; 95% CI, 0.82-2.66; *P* = .20).

Among 300 patients with baseline frailty (CSHA-CFS scores of 5 or higher), the intervention reduced postoperative delirium risk compared with those in the control group (OR, 0.62; 95% CI, 0.41-0.95; *P* = .03). The benefit was significant in 260 frail patients undergoing noncardiac surgery (OR, 0.67; 95% CI, 0.50-0.89; *P* = .005). The association between the intervention and delirium was not significant in 40 frail patients undergoing cardiac surgery (OR, 3.01; 95% CI, 0.14-63.33; *P* = .48).

Compared with patients in the control group, patients in the intervention group experienced fewer delirium days (523 vs 699 days; mean, 0.7 vs 1.0; mean difference, 0.3 days; 95% CI, 0.1-0.6; *P* = .03; n = 1457) and lower percentage of days with delirium (5.3% vs 6.9%; *P* = .03) (Table 4); once delirium occurred, its length in days was no different between groups (median, 3 days; *P* = .84) In patients undergoing noncardiac procedures, delirium days (171 vs 310 days; mean, 0.4 vs 0.7; mean difference, 0.3 days; 95% CI, 0.1-0.5; *P* = .006; n = 929) and percentage of days with delirium (3.0% vs 4.7%; *P* = .007) were significantly lower in the intervention group compared with the control group. No differences were identified in patients undergoing cardiac procedures.

The mean length of stay was significantly lower in the intervention group compared with the control group (11.1 vs 11.4 days; *P* = .01) (eTable 7 in Supplement 2). This benefit was significant in those undergoing cardiac procedures (10.7 vs 11.2 days; *P* = .046; n = 528) but not in those undergoing noncardiac procedures.

Fewer postoperative transfers to a rehabilitation hospital (intervention group: 336; control group: 410) occurred following intervention (χ^2^_1_ = 16.54; *P* < .001). The intervention influenced postoperative medication requirements. In patients undergoing noncardiac procedures, opiate administration (χ^2^_1_ = 21.76; *P* < .001), benzodiazepines (χ^2^_1_ = 11.49; *P* = .001), and newly dispensed neuroleptics (χ^2^_1_ = 6.94; *P* = .008) were reduced in the intervention group compared with the control group. For those undergoing cardiac procedures, only postoperative opiate administration decreased (χ^2^_1_ = 33.74; *P* < .001).

Most patients undergoing cardiac procedures (intervention group: 152; control group: 200) spent at least 1 postoperative day in the intensive care unit (χ^2^_1_ = 27.03; *P* < .001). No difference in intensive care unit length of stay was found between groups (*t*_350_ = 1.53; *P* = .13), a caveat being that one center routinely monitored postoperative patients in intensive care unit for 3 days. The intervention caused no adverse events. Falls, strokes or brain hemorrhage, hemorrhage, embolism, or thrombosis, death, and surgical reintervention were similar in both groups.

## Discussion

The clinical delirium syndrome is the final common pathway for many pathophysiological conditions. The significant baseline cognitive frailty we describe in this population is compounded by the physiologic stress and various biochemical cascades inherent to the perioperative and surgical context. The individual’s vulnerability and delirium risk profile,^12,19,20,21,22^ combined with surgery, make prevention in this population a challenging task. Our daily multicomponent intervention significantly reduced the relative risk of delirium by 33.2% and its duration by 139 days overall (171 vs 310 days with delirium; mean, 0.4 vs 0.7 days; *P* = .006) in patients undergoing many types of surgical procedures. It also reduced potentially harmful pharmacological exposure.

Earlier delirium prevention or duration reduction studies targeting smaller, more homogeneous populations either showed no effect, higher delirium rates with intervention,^36^ or lesser improvements in delirium prevalence.^12^ To our knowledge, none evaluated preoperative risk, frailty, or included effect on various medications.

Little evidence supports the effectiveness of currently recommended multicomponent delirium prevention approaches.^15,18^ A systematic review^24^ describing nonpharmacological delirium prevention in surgical patients included 3 hip fracture trials in its meta-analysis, suggesting elective surgery requires a clearer definition. A Taiwanese center^37^ assessed delirium prevention in patients undergoing abdominal procedures using cluster methodology resembling ours. Specialized nurses’ daily prevention based on 3 HELP modules reduced delirium by more than 50% (RR, 0.44; 95% CI, 0.23-0.83). In contrast, a Duke University pre-post study led by surgeons keenly interested in risk-reduction in intraabdominal surgery perioperative periods improved length of stay, shock, and ileus rates but worsened delirium rates.^36^

To our knowledge, this trial is the largest multicenter study showing effective delirium prevention in elective surgery in older adults and the only one to examine a wide range of surgical procedures. Our AKTIVER delirium prevention program combined evidence-based best practice components ranging from preadmission risk-stratifying ongoing assessments and follow-up at hospital discharge. We combined strategic education and knowledge dissemination techniques with daily prevention. To these interventions, adapted to individual needs as described in medical settings, we added caregiver presence, reassurance to minimize anxiety, and the provision of humane support in unknown surroundings, based on the German The Older Person in the Operating Room model.^43^ As in other examples of delirium prevention bundles,^62^ pain management, medication reduction, and human interactions were addressed daily and systematically by nurse specialists during daily rounds. Our experts mentored the trained volunteers who provided AKTIVER module care daily, tailoring it to individual needs and family engagement. Our ability to integrate diverse AKTIVER activities in previously inexperienced centers within the 6 weeks of operationalizing the study and our intervention fidelity metrics speaks to its feasibility. A consistent benefit was shown across different groups of vulnerable older surgical patients. Since AKTIVER delirium prevention integrated volunteers and family members, intervention delivery consistency is maintained while minimizing costs.

We controlled for various risk factors through extensive presurgical phenotyping of multidimensional clinical parameters and cognitive status. The delirium rates we observed (21.6% overall, 35.7% in those undergoing cardiac procedures, and 13.6% in those undergoing noncardiac procedures) were similar to other reports.^11,15,34,63^ Our elderly patients (age range, 70 to 98 years) represented high-risk populations with cognitive deficits, multiple comorbidities, and frailty. Including neuropsychiatric diseases maximizes our findings’ generalizability.^23^ As hospitals grapple with elective surgery delays because of the diversion of resources owing to the worldwide COVID-19 pandemic, reducing postoperative complications in older patients appears timely. Most interventions in our cohort, albeit elective (eg, colon cancer requiring resection), required rapid surgery.

Hospital-based professionals were supported by trained volunteers and aides, a potentially useful resource during the COVID-19 pandemic and postpandemic periods, which strained worldwide health care resources. Patients, health care workers, and relatives all benefit from delirium prevention, as delirium burdens them all.^64^ We are analyzing hospital and long-term care costs and benefits to identify the economic benefits of this delirium prevention approach.^65^ Whether pairing our approach with a prehabilitation stimulation program^66^ would further reduce delirium incidence merits testing.

### Limitations

The following limitations should be considered. Our intervention had no effect on delirium occurrence in patients undergoing cardiac surgery. To our knowledge, no publication describes effective nonpharmacological delirium prevention in this population. A small pilot study^67^ showed no effect on delirium severity. Pharmacological agents, notably dexmedetomidine, may prevent delirium after cardiac surgery^63^ and in the critically ill^68^ but require hemodynamic monitoring. Risk factors common in cardiac surgery (age, low ejection fraction, kidney insufficiency, atrial fibrillation, and vasculopathy)^69^ are unmodifiable, and blood-brain barrier disruption^70^ characterizes delirium in older patients after cardiac surgery. Bispectral index-guided anesthesia and modulating cerebral oxygenation might be protective.^71^ The effectiveness of perioperative physiological neuromonitoring-driven interventions further differentiates delirium prevention after cardiac vs noncardiac surgery. These findings and our study’s dichotomous results suggest the postoperative delirium pattern in cardiac surgery differs from general surgery postoperative delirium in risks, potential interventions, and outcomes.

The feasibility and effectiveness of this intervention should be tested, especially in smaller and nonacademic hospitals and in rural areas. Patient readmissions after hospital discharge were not documented and would have added valuable outcome-related information. Intensive care unit stay analyses must be interpreted cautiously, as hospital protocols differed in the clusters. The temporal change sensitivity analysis in our models suggests that 12-week intervals suffice to ascertain the effect of our intervention. We hope qualitative researchers will capture family and patient experience in future studies. Ongoing quality assurance initiatives could identify reproducibility in different medical cultures and languages.

## Conclusions

The PAWEL trial successfully demonstrated that a structured, reliably reproducible, and safe nonpharmacological delirium prevention method is effective. The PAWEL protocol integrates a rigorously protocolized approach with individual patient’s needs. Its implementation significantly reduces postoperative delirium risk in patients 70 years or older undergoing noncardiac procedures. These results suggest that this delirium prevention program benefits patients undergoing elective general surgical and orthopedic procedures.

## Notes

Supplement 1. Trial protocol.

Supplement 2. **eTable 1.** Baseline characteristics of participants in each centre. **eTable 2.** List of surgical procedures performed. **eTable 3.** Missing data for the main study variables. **eTable 4.** Delirium rate (%) per period in each centre. **eTable 5.** GEE analysis (model 2). **eTable 6.** GEE analysis (model 3) of cardiac and noncardiac surgery group. **eTable 7.** Effect of the intervention on overall delirium duration.

Supplement 3. The PAWEL Study Group.

Supplement 4. Data sharing statement.
