# Supplementary material for: Outcomes of a Delirium Prevention Program in Older Persons After Elective Surgery: A Stepped-Wedge Cluster Randomized Clinical Trial
Source: JAMA Surg. 2021 Dec 15;157(2):e216370. doi: 10.1001/jamasurg.2021.6370 (PMC8674802; doi:10.1001/jamasurg.2021.6370)
Supplement: Supplement 1. — Trial protocol. [file jamasurg-e216370-s001.pdf]

Grant Application

PAWEL: Patientensicherheit, Wirtschaftlichkeit und Lebensqualität: Reduktion von Delirrisiko und POCD im Alter

PAWEL: Patient safety, cost-effectiveness, and quality of life: reducing delirium risk and POCD after elective surgery in older adults.

Interventional Care Research Study Version 07 2016/08/16/	
Project management or consortium leadership	Prof. Dr. med. Gerhard Eschweiler, Geriatric Center (GZ) at the University Clinic (UK) Tübingen (project management), Prof. Dr. phil. Dr. med. Michael Rapp University of Potsdam (methodology)
Participating institutions/ cooperation partners	**UK Tübingen:** Geriatric Center (GZ), commercial directorate, nursing management, cardiac surgery, orthopedics, anesthesia/ **Klinikum Stuttgart** with geriatric psychiatry, GZ, vascular surgery, trauma and abdominal surgery, orthopedics, anesthesia, advanced nursing training/ **GZ of the University hospitals Ulm and Freiburg** as well as **GZ Karlsruhe** with cardiac surgery, orthopedics, anesthesia/ **Mrs. Prof Joos**, General Medicine Tübingen and family physicians/ **Prof. M. Rapp**, University of Potsdam/ **Prof J. Wasem** University of Duisburg-Essen /**AOK Baden-Württemberg**, Care Management Department
Project title/ acronym	PAWEL: Patient safety, cost-effectiveness, and quality of life: reducing delirium risk and POCD after elective surgery in older adults.
Subject area	Further development of quality assurance and patient safety in the care of older adults undergoing elective surgery through trans sectoral delirium and POCD prevention.
Research field/ disease/ target population	Trans sectoral care, delirium prevention, postoperative cognitive dysfunction (POCD), dementia, older adults, elective surgery, quality improvement, cost-effectiveness.
Project goals/hypothesis	Development of prehospital risk screening and trans sectoral multimodal intervention in patients > 70 years prior to elective surgery 1) to reduce delirium prevalence by 40% through multimodal perioperative delirium prevention 2) to reduce POCD after 6 months by 20% 3) to reduce associated health care costs for statutory health insurance (SHI) and hospital providers. 4) Implementation strategy for routine care (identification of barriers/ enablers; development of patient pathways, training and information materials, dissemination pathways).
Study type and methodology	Stepped wedge design with cluster randomization of 10 surgical departments for evaluation of multimodal delirium and POCD prevention intervention with 5 consecutive measurement time points pre-admission, preoperative, postoperative, and 2 and 6 months postoperatively. Long-term follow-up after 12 months. Trans sectoral care structure analysis. Qualitative survey pre- and postintervention, knowledge and training evaluation.
Intervention	Development of delirium risk score: Recruitment and pre-hospital delirium risk assessment for elective chirurgical procedures (orthopedic, joint replacement, cardiac and vascular surgery) in elderly patients in cooperation with referring physicians and surgical outpatient clinics. **Intervention:** three-stage training on delirium diagnostics, prevention, management and cognitive dysfunction (physicians, nurses, physiotherapists, medical aides (MFAs); Qualification and implementation of operating room companions

	(students, geriatric nursing aides); and preventive teams (physicians, nurses, volunteers) for trans sectoral multimodal delirium management in delirium risk patients (7 modules), pre- and perioperative until discharge. Trans sectoral cooperation (general practitioners, referring physicians, MFA) for guideline-compliant patient management and avoidance of inadequate medication.
Study population and sample size	10 surgical departments with 4300 patients (> 70 years) in orthopedics, abdominal, cardiac and vascular surgery, including anesthesia and GZ; for the development of the delirium risk score (N=1800 patients); in the intervention phase (N=1500 patients), thereof approx. 50% SHI patients of one German health insurance (AOK); with 25 patients per quarter per department. Qualitative survey: 10 focus groups (1 per site before/after intervention) with referring GPs and specialists.
Database	**For delirium risk score:** physical risks, whisper-, visual acuity test, Charlston Comorbidity Score, sniffin' sticks, anticholinergic load, delirium/dementia history, IQCODE, medication amount, PSQI, ESS, FIM, PHQ-9, SF-12, Apache II, operation and clinical data, I-CAM, NuDesc, burden of care (N=1800 patients > 70 years).<br>**For intervention comparison:** (N=1500 patients undergoing elective surgery > 70 years).<br>**Primary outcome:** delirium prevalence measured by daily delirium screening (I-CAM/ NuDesc) over 7 days post-op, and at 2 and 6 months.<br>**Secondary outcomes:** 1) prevalence of POCD measured by at least 0.5 standard deviations worsened cognitive performance skills in one of the following tests at 2- or 6-months post-surgery: MoCA, TMT A/B, digit span.<br>2) multivariate change in the presented cognitive test battery over 2 and 6 months. For planned analyses: SF-12, Care Giver Burden, EQ5D, FIM, MAI, risk indicators. For planned health economic analyses: Hospital costs for two centers; AOK costs for all AOK patients over 24 months (12 months pre-surgery and 12 months post-surgery); cost analysis for ambulatory and inpatient assessments, trainings, costs of OR companions, preventive team. For process analyses: knowledge assessment before/after training, degree of implementation of intervention related to training, OR companions, preventive teams and post-inpatient delirium management in all centers. Qualitative survey: focus group transcripts (n=10).
Data Analysis	**primary outcome:** logistic regression analyses with cluster adjustment.<br>**secondary outcomes and planned analyses:** additional mixed linear and logistic regression models with fixed and random factors to estimate heterogeneity across clusters.
Target variables	**Primary and secondary outcomes for the intervention:** prevalence of delirium, prevalence of POCD, multivariate change in cognitive skills (MoCA, TMT A/B, digit span, SF-12).<br>**Health economic analysis:** hospital costs, post-inpatient costs incl. rehabilitation and SHI costs of outpatient and inpatient care (AOK data, 50% of patients), provider costs of the hospital stay (regular ward, IMC, ICU) and length of stay (routine hospital data).
Requested funding period	3 years 1/1/2017-12/31/2019 (WP 8 Health economic analysis until 9/30/2020).
Requested funding	6.26 million euros - 2.09 million euros per funding year

2 Applicants

Responsible persons / persons involved

Name	Institution	Phone, fax, e-mail	Responsibility/ Role
Prof. Dr. med. Gerhard Eschweiler	Geriatric Center at the University Hospital, Tübingen	Phone: 07071-298-5222, Fax- 4628 gerhard.eschweiler-@med.uni-tuebingen.de	Consortium management, project management, clinical coordination
Prof. Dr. med. Dr. phil. Michael Rapp	Department of Social and Preventive Medicine, University of Potsdam	Phone: 0331 977-4095 Fax: -1078 mrapp@uni-potsdam.de	Study design and biometry
Prof. Dr. Jürgen Wasem	University of Duisburg-Essen, Essen Campus, Chair of Medical Management	Phone: 0201 183-4283 Fax: 0201 183-4073 juergen.wasem@uni-due.de	Health Economics
PD Dr. med. Christine Thomas	Department of Old Age, Geriatric Psychiatry and Psychotherapy, Klinikum Stuttgart	Phone: 0711/27822802 c.thomas@klinikum-stuttgart.de	Clinical coordination Stuttgart, Qualifications and trainings of Interventions
Prof Dr.med. Stefan Klöppel	Center for Geriatrics and Gerontology, University Medical Center, Freiburg	Phone: 0761 270-70980 stefan.kloeppel@unikli-nik-freiburg.de	Clinical coordination Freiburg
Prof. Dr. med. Christine von Arnim	Department of Neurology, University Hospital, Ulm	Phone: 0731/500-63011 Fax: 0731/177-1202 Christine.arnim@uni-ulm.de	Clinical coordination Ulm
Dr. Brigitte R Metz	Geriatric Center Karlsruhe, ViDia Christian Clinics Karlsruhe, Karlsruhe	Phone: 0721-8892701 Fax 0721-889-3912 b.metz@diak-ka.de	Clinical coordination Karlsruhe
Prof. Dr. Uwe Mehlhorn	HELIOS Clinic for Heart Surgery Karlsruhe	Phone: +491729738-131 Fax: +49-172-9738-119 uwe.mehlhorn@helios-kliniken.de	Cardiac surgery recruitment
Prof. Dr. Stefanie Joos	Institute for General Practice and Interprofessional Care, University Hospital Tübingen, Tübingen		Clinical coordination of outpatient care
Ms Ramona Auer (M.A.)	Allgemeine Ortskrankenkasse (AOK) Baden-Württemberg, Stuttgart		Health economic data of the health insurance company

Acknowledgement:

It is confirmed that the project outline has not been submitted for any other funding.

3 Project goals

Research question and working hypothesis

Delirium is associated with increased morbidity and mortality, cognitive impairment, dementia progression, and institutionalization rates (Witlox, Eurelings et al. 2010). Delirium can be assessed validly with the Confusion Assessment Method (I-CAM) (Thomas, Kreisel et al. 2012). Elective surgery in the elderly aims to achieve a better quality of life while maintaining cost-effectiveness. We investigate (a) to what extent a trans sectoral multimodal delirium prevention intervention additionally improves quality of life by reducing delirium prevalence and cognitive deficits for patients over 70 years of age undergoing elective surgery, and (b) whether this intervention is cost-effective in the German health care system - both from the perspective of the health service provider and from the of the health insurer's perspective.

Our trans sectoral longitudinal study in a stepped-wedge design over 30 months in 1500 patients with elective surgery aims to show that

(1) a delirium reduction of at least 40% compared to baseline is achieved by the multisectoral, multimodal, and multidisciplinary intervention; and

(2) a 20% reduction in post-operative cognitive dysfunction (POCD) compared with standard care can be expected.

(3) the trans sectoral intervention including the delirium risk identification required for this purpose (delirium risk score), team qualification and non-pharmacological prevention module provision is cost-effective for the service provider, so that with increased quality of outcome no additional costs arise, care burdens and care requirements are lower than after standard treatment.

In addition, a delirium risk score for elective surgery in the elderly will be developed in the first consecutive 750 patients and validated in the following 750 patients (in departments without intervention), the delirium prevalence and POCD prevalence will be assessed for the first time multicenter in a large German cohort after 6 and 12 months depending on the delirium risk (among others), a team qualification and training concept will be developed, implemented and evaluated in hospitals and medical practices, and potential delirium complications and their costs will be recorded.

Scientific and structural concept

The occurrence of postoperative delirium (POD) is largely dependent on preexisting delirium-promoting factors such as age, preexisting brain damage and dementia, cognitive, sensory, and mobility deficits, multimorbidity, and polypharmacy (Guenther, Riedel et al. 2016). A significant proportion of delirium is preventable if delirium risk factors are taken into account (RR 0.69, 95% CI 0.59 - 0.81) (Siddiqi, Harrison et al. 2016). Delirium is therefore a quality indicator for age-appropriate therapy, and delirium prevention is an essential parameter of patient safety (Inouye 2006, Reston and Schoelles 2013). Early indicators of cognitive deficits (hyposmia (Brown, Morrissey et al. 2015), sleep (Todd, Gellrich et al., Patel, Baldwin et al. 2014), and subjective memory impairment (SMI) (Hagen, Ehlis et al. 2015) (Jessen, Wolfsgruber et al. 2014)) are also delirium-relevant and will be integrated here for the first time into a delirium risk score for elective surgery. The perioperative phase with its stresses due to anesthesia, surgery-related factors, pain, and immunological activation is a major trigger of POD (Guenther, Riedel et al. 2016). Post-operative cognitive dysfunction (POCD) (Monk and Price 2011, Rundshagen 2014), which often occurs after POD, needs to be related to preoperative abilities in order to quantify the extent of delirium sequelae (Rudolph, Inouye et al. 2010) especially in

terms of dementia development (Fong, Davis et al. 2015) and to determine its health economic costs. POD and POCD are associated with higher mortality and postoperative complications (such as infections, decubiti, incontinence (Rundshagen 2014), prolonged length of stay (Visser, Prent et al. 2015), intensified care and therapy (Pretto, Spirig et al. 2009), increased need for long-term care and dementia, and increased health and long-term care insurance costs (Gleason, Schmitt et al. 2015, Guenther, Riedel et al. 2016). POCD is only partially reversible and, like POD, is an independent modulator of progressive dementia (Fong, Jones et al. 2009).

Current guidelines on POD management (American Geriatrics Society Expert Panel on Postoperative Delirium in Older Adults 2015) emphasize the importance of delirium prevention. A multimodal nonpharmacologic approach (Bringemeier, Thomas et al. 2015) is considered the ideal way (Zhang, Lu et al. 2013, Siddiqi, Harrison et al. 2016). Evidence-based models (Bakker, Persoon et al. 2014, Patel, Baldwin et al. 2014, Bringemeier, Thomas et al. 2015, Hshieh, Yue et al. 2015) achieve a delirium risk reduction of 53% (95% CI, 0.38 to 0.58) in a meta-analysis (Hshieh, Yue et al. 2015), but so far do not include the ambulant sector. The cross-sector delirium prevention model planned here extends the inpatient models to include perioperative monitoring (Gurlit and Mollmann 2008) and prehospital risk reduction counseling ("prehabilitation"). In elective surgery, pre-stationary primary delirium prevention (Yang, Inouye et al. 2008) is possible, and improved age-sensitive care (e.g., FORTA criteria (Wehling, Burkhardt et al. 2016)) is sustainable. Thus, an even more effective delirium reduction can be expected from a transsectoral, multimodal prevention approach, which should also have an impact on POCD prevalence at 6 months.

In a network of 3 university and 2 tertiary care clinics (with 5 geriatric centers and 20 departments) the delirium risk and delirium assessment should be optimized, a trans sectoral delirium prevention intervention should be established and evaluated regarding effectiveness and cost efficiency. This involves evidence-based delirium diagnosis, neuropsychological testing, and multimodal multiprofessional intervention and follow-up over 6 months for POCD and 12 months for dementia development.

4 Contribution to the improvement of care

4.1 Relevance

Patient safety is significantly compromised by POD. The delirium rate is considered a quality indicator for age-sensitive hospital care. Morbidity and mortality are significantly increased after POD in the short and long term, and persistent POCD reduces daily living skills and autonomy, significantly impairing quality of life. The incidence of POD and POCD increases exponentially with age and is dependent on medical staff's delirium expertise. Dependency of care, institutionalization, and dementia development are of considerable relevance to health economy. The current GHOSt study found a 40% prevalence of cognitive impairment in patients over 70 years in acute care hospitals in Baden-Württemberg and Bavaria. Of those, half had mild cognitive impairment and half had dementia. In less than half of the patients their cognitive impairment was known to the attending physicians and nurses, suggesting a significant diagnostic deficit in German hospitals (Grund et al., 2015). Multicenter care research to evaluate the additional effort therefore is urgently needed (Gurlit, Kratz et al 2016).

4.2 Improving care

This nationally unique approach will:

1) assess the incidence of POD and POCD after 2, 6, and 12 months in the actual reality of healthcare delivery in about 1500 patients over 70 years of age after elective surgery (heart, vascular, joint replacement, spine) in a multicenter design.

2) develop and validate a pre-surgical delirium risk score for patients over 70 years of age, based on a pre-hospital cognitive assessment that can be implemented into routine care.

3) implement a cross-sectoral, individualized, multi-professionally supported multimodal delirium and POCD prevention program and evaluate it in terms of health economics.

4) assess he implementability of medication changes based on the FORTA criteria in the pre-anesthetic and perioperative settings.

5) evaluate the implementability of the pre-stationary and post-stationary cognitive assessment by medical aides (MFA) in primary care practices and the necessary preconditions.

The results should form the basis for German guidelines on delirium prevention and management (so far only available for intensive care patients) in surgery and guidelines on the management of the frequent occurring but insufficiently defined POCD. This will secure the positive outcome of elective surgery, improve patient safety and quality of life, and minimize a longer-term dementia risk. Patients with dementia carry the highest risk of POD, will therefore benefit disproportionately from the project and are thus purposefully included (with the consent of their authorized representative/caregiver). A local ethics board will be consulted in case of doubt. Patients with dementia and POD increase the demands on staff and resources and have a longer length of stay after surgeries; therefore, in addition to patients and relatives, hospitals are also interested in identifying cognitive deficits at an early stage and minimizing POD and POCD. A health economic evaluation will prove the cost-effectiveness of the intervention from the perspective of the hospitals (microeconomic) and from the perspective of the health care and long-term care insurance company AOK-Baden-Württemberg with regard to inpatient and outpatient costs in the first year postoperatively.

4.3 Innovation content of the project

This multicenter intervention study builds on the applicants' numerous preliminary studies on dementia and delirium in different inpatient settings. Unique is the trans sectoral approach with 12-month follow-up with regard to the long-term consequences of delirium in Germany. The approach has several objectives:

1) Further development of a clinically meaningful delirium risk score to estimate the delirium risk of older people at risk and collection of valid perioperative delirium and post-inpatient POCD incidences over 6 months and 12 months for the most common elective surgeries.

2) Evaluation of a new, cross-sectoral, multimodal delirium and POCD prevention intervention with high effectiveness in the actual German health care realities, which is maintained multi-professionally and inter-disciplinarily. The baseline assessments are performed prior to elective surgery, as only in this occasion a pre-hospital cognitive status can be assessed validly as a baseline for the detection of potential post-operative cognitive decline. The evaluation effort is very high in terms of personnel, as it involves the cooperation of 5 hospitals with geriatrics, anesthesiology, surgery (heart, vessels, bone), geriatric psychiatry as well as nursing education, management, and research. For the first time, trans

sectoral, transdisciplinary, and transprofessional data can be collected and evaluated scientifically from an economic and health care science perspective.

3) Cost-effectiveness calculation of the preventive effort and the economic long-term effect, taking into account the training costs, intervention costs, savings through complication avoidance and reduced length of stay, as well as long-term reduction of care costs in Germany. For the participating patients insured by the AOK Baden-Württemberg (approx. 50% of the total), trans-sectoral costs for physician visits, medications, rehabilitation, and nursing care measures up to month 12 postoperatively are recorded for the first time and compared to the avoided POCD cases. For the first time, the costs and savings during the initial hospitalization are determined (and compared with the outcome after standard treatment) also from the perspective of 2 different hospital institutions (a university hospital and a large municipal hospital "Klinikum Stuttgart"). The nursing care services, and its overall costs are also recorded before and during the intervention, to gain knowledge about the nursing care demand of these high-risk patients. Thus, potential additional remuneration for delirium patients in the surgical setting or those with high delirium risk may be calculated for the first time. A transfer of these results to primarily delirious patients (at admission) in emergency medicine, internal medicine, geriatrics, and other surgical and conservative specialties is obvious.

5 Qualification and previous experience of the proposers

The participating hospitals and geriatric centers have been conducting research as a network for many years to improve quality of care in elderly, geriatric, and psychiatric patients (see CVs).

The **Geriatric Center Tübingen** (GZT, head of office: Prof. Dr. Eschweiler) is a cooperation of five institutions and participates in several research networks (e.g. Lebensphasenhaus, TREND-Studie, Study on the effectiveness of blue LED light (BMBF funding NiViL 2015 to 2018)) on geriatric topics. Furthermore, there are projects to support caring relatives (GZT: BMBF 2012-2015, TABLU in cooperation with Red Cross and Altenhilfe (Elderly Care) Tübingen and KRISTINA (EU project (2016-2018) on assistance by means of an expert system). At the **Klinikum Stuttgart** (PD Dr. Thomas) there is extensive expertise in delirium screening, in development and implementation of multimodal, multi-professional delirium prevention programs (twice funded by the Robert Bosch Foundation; involvement in collaborative projects of the European Delirium Association and guideline development (e.g. geriatric traumatology) and in the scientific evaluation of delirium prevention models (Robert-Bosch-Stiftung, HuberTDa). At the **Center for Geriatrics and Gerontology of the University Hospital Freiburg (ZGGF),** Prof. Dr. T. Klöppel has established a local delirium standard and has been active for many years in health care research projects funded by the BMBF (e.g., home-based daily life training for patients with dementia, depression treatment). In the ZGGF, numerous non-pharmacological studies on the delay of cognitive decline in dementia of the Alzheimer's type and mixed type were successfully conducted (everyday cognitive training in dementia: BMG, lighthouse project WHEDA, 2008-2010; DFG, REDALI-DEM 2011-2015 under the leadership of the ZGGF, Dr. Voigt-Radloff, Prof. Hüll). ZGGF: BMBF 2012-2015, FABEL in cooperation with the Kath. Hochschule Freiburg**). The Institute of Social and Preventive Medicine at the University of Potsdam** (Prof. Dr. Dr. Rapp) has many years of expertise in the implementation and evaluation of guideline implementation studies and evaluation studies of new therapeutic procedures in elderly patients with geriatric psychiatry clinical disorders. The Chair of **Medical Management at the University of Duisburg-Essen** (Prof. Dr. Wasem) is a leading senior scholar in the development and implementation of methods for studies in the areas of benefit evaluation, cost-benefit evaluation, health technology assessment and health services research in Germany. Dr. Metz is head of the **Geriatric Center Karlsruhe** and has been chairwoman of the State Geriatrics Working Group for more than 10 years. She has been involved in numerous medical care

projects in Germany (e.g. the dementia project WHEDA in cooperation with the centers in Freiburg and Tübingen). The **Geriatric Center Ulm** (GZU Ulm) is an association of several hospitals (including geriatrics, neurology and orthopedics) and is well connected since years through several joint projects. Prof. von Arnim (head of the GZU office) has been active in clinical projects on dementia for many years as head of the memory clinic (e.g. WIN study funded by the Heidelberg Academy, Ulm Lighthouse Project Dementia BMG). Prof. Denkinger (Chairman of GZU) is involved in several geriatric care studies (e.g. EU-funded SITLESS and SHELTER studies, joint project: "Medication and life situation in old age"). Prof. Reichel is president of the German Society for Orthopedics and Trauma Surgery (DGOU) and the German Society for Orthopedics and Orthopedic Surgery (DGOOC). In a population-based longitudinal study in older adults (ActiFE; currently funded by DFG), they are investigating the role of physical activity for successful aging together with the Epidemiology Department of the University of Ulm.

6 Scientific and methodological approach: Project Implementation

The study is conducted as a prospective, randomized, controlled intervention trial in 1500 elderly patients at increased risk of delirium:

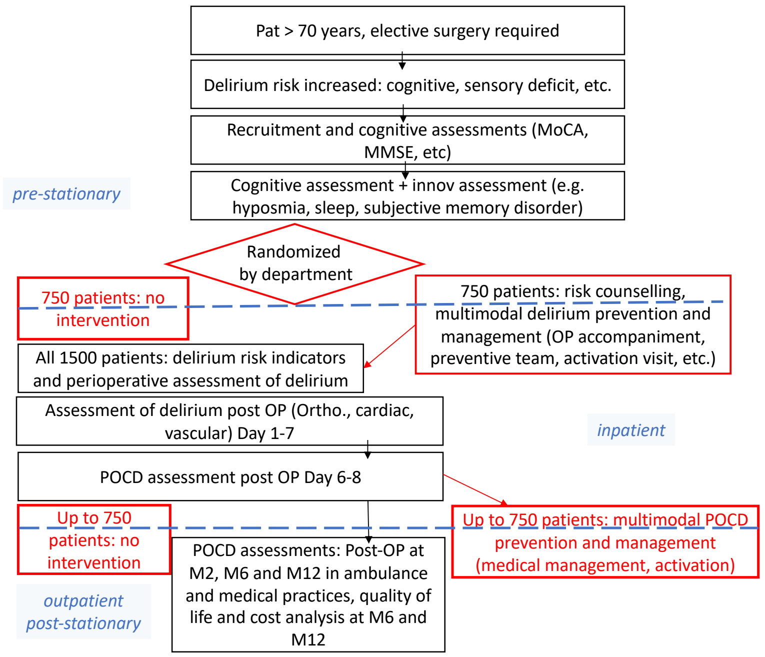

The health care study is structured into 9 work packages (WP) (see Table 1).

WP 1: Prehospital assessment and risk identification (from Q2).

Quantitative: prehospital evaluation of delirium and POCD risk ((Inouye 2000, Rudolph, Jones et al. 2007, Oh, Li et al. 2015, Guenther, Riedel et al. 2016) as a screening (4 factors): cognitive impairment, visual impairment, multimorbidity or mobility impairment, renal insufficiency, and 2 newly documented risk factors in Germany (Hessler, Bronner et al, 2015): current cigarette use (Hessler et al, 2015) and alcohol consumption (> 3 drinks/day).

Inclusion: patients over 70 years of age undergoing elective surgery.

Exclusion criteria: Newly discovered severe dementia (MMST < 15, MoCA < 8 p.) without a substitute decision-maker, then exclusion from study and referral to Memory Clinic for further care, insufficient German language skills, unstable vital signs.

Assessment tools: Medical history (plus third-party anamnesis and medical record review, if applicable) of cognitive, emotional, and circadian abnormalities (dementia, MCI, depression, sleep disorder, sleep medication use, current cigarette use, and delirium in the past) (15 min), IQCODE (Jorm 1994) by primary caregivers, PHQ-9 by patients, clinical examination of sensory impairments (whisper and visual acuity test) and neurological abnormalities (e.g. focal symptoms, Parkinson symptoms), Charlson Comorbidity Index (Charlson, Pompei et al. 1987), amount of necessary daily care (in degrees). MOCA (10 minutes), Digit Span Backwards, Trail Making Test A and B (6 minutes) as a measure of executive functioning and attention shifting (30 minutes), SMI Subjective Memory Impairment (1 minute), Quality of Life EQ-5D (1 min), SF-12 Quality of Life (10 minutes), Circadian Rhythm and Sleep Behavior: Chronotype determination, (1 min), Epworth Sleeping Scale (ESS) by pat. (1-2 min), olfactory function: Sniffin´ Sticks 12 version (10 min), medication recording and evaluation according to FORTA criteria (Wehling, Burkhardt et al. 2016) (from medication schedule in consultation with primary care physician).

Qualitative approach: Transsectoral analysis of perioperative patient pathways

The analysis of the perioperative patient pathways is performed in order to adapt the intervention to the intersectoral everyday care and to enable or facilitate the subsequent implementation in routine care. For this purpose, focus groups with primary care physicians, referring physicians and a participant of the geriatric center will be conducted (one focus group per site and per topic; a total of 10 focus groups with approx. 10 participants each with explorative questions about the need for and sensitivity to delirium/ POCD, professional knowledge about delirium, dementia, depression, and other aspects).

To ensure the feasibility of implementation, the results will be disseminated in workshops at 1. a training event, 2. at the annual “Dementia Day” (in cooperation with the District Medical Association of Baden-Württemberg), 3. at the “Day of General Medicine” at the Institute of General Medicine and Interprofessional Care in Tübingen, and the development of an integrated care pathway, including the necessary suitable information tools (information flyers), and training modules for physicians and MFA. In addition, the possibility to delegate POCD assessments to trained MFAs will be tested. In routine care, especially postoperative observation for POCD prevention will take place in the outpatient sector. In this context, primary care physicians will play a central role. In the sense of an innovative interprofessional approach, it will be tested which tasks could be delegated to trained MFAs. In the study phase, the POCD assessments will be performed by MFAs and nurses in the outpatient departments of the geriatric centers.

WP	WP Leader	Work and schedule in project months											
	Centre	Study year 1				Study year 2				Study year 3			
		Q1	Q2	Q3	Q4	Q5	Q6	Q7	Q8	Q9	Q10	Q11	Q12
		Month 1-3	Month 4-6	Month 7-9	Month 10-12	Month 1-3	Month 4-6	Month 7-9	Month 10-12	Month 1-3	Month 4-6	Month 7-9	Month 10-12
WP 1 Delirium risk pre-stationary	Tübingen	Preparation and assessment training	Pre-stationary risk of delirium: physical risk, Whisper-Test, visual test, Charlson Comorbidity Index, Sniffin sticks, Anticholinergic Load, delirium / dementia history, IQCODE, MAI, number of medications, PSQI, ESS, FIM, PHQ-9, SF-12, Apache II, OP- and clin. data, I-CAM, NuDesc, care burden										Publications, final report
WP 2 Perioperative delirium assessment	Stuttgart	Preparation and assessment training	Perioperative delirium assessment day 1-6 CAM and to T2 and T6 NuDesc						Training courses MFAs and Nurses				Publications, final report
WP 3 Post-stationary assessment	General medicine Tübingen	Assessment training	Post-stationary assessments postM2, postM6 and postM12 in outpatients' clinics, medical practices and, if necessary, in family, focus groups, MFAs training courses										Evaluations, Publications, Dissemination
WP 4 Training	Stuttgart	Preparation	Knowledge-based multiprofessional	Team training in intervention departments including anesthesia and MFAs as required					Blended Learning: assessment, management and prevention of delirium				Publications, final report
		Study Nurse + Doctors	Eval. training courses, intervention counselling and module training						Data analysis				
WP 5 Multimodal delirium prevention	All Centres		Intervention preparation	Multimodal prevention and management of delirium (7 modules)									Publications, final report
WP 6 Data management	Potsdam	Preparation of the database	Data input, data sharing										
WP 7 Evaluation	Potsdam	Preparation of statistical models	Information, recruitment	Detection of delirium risk, prevalence, severity and duration of the delirium for 7 days, etc.					Analyses post OP: MoCA, TMT A / B, digit span. Multivariate modification of the presented cognitive test battery over 2 and 6 months. For				Publications, final report

					planned analyses: SF-12, Caregiver Burden, FIM, Risk indicators	
WP 8 Economics	Essen	Preparation, modelling	Micro-level incomes of the 2 clinic Tübingen and Stuttgart		Data analysis of AOK patients: outpatient costs, rehabilitation, medication, remedies, care and re-hospitalization	Publications, final report
WP 9 Consortium management, monitoring, ethical	Tübingen	Consortium agreements, employees	Ethics application, materials	Project coordination, monitoring		Dissemination
Student assistants, nursing assistants, etc.	All partner	Operation companion, delirium companion, office activities, data maintenance and input				

WP 2: Perioperative delirium assessment

During the basic assessment phase (control period), a geriatric checklist from the geriatric concept of Baden-Württemberg (GC), FIM (Functional Independence measure), NRS (Nutrition Rating score), cognitive short test, delirium screening are performed at the time of hospital admission, and cognitive short test, and barthel-index are applied at discharge. Postoperatively, a daily delirium assessment is performed by CAM by study nurse or study physician. In the intervention, a daily delirium assessment (CAM/4AT/Nudesc) (by raters (nurse) will be performed. For this purpose, standardized delirium diagnostics are established and trained on the pilot wards (4AT/NuDESC by ward nurses, in case of positive findings I-CAM by physicians and specially trained psychogeriatric nurses) for early delirium detection. In addition, standards for premedication, surgical and anesthesia adjustment, pain level therapy, infection therapy, drug-induced sleep and age-appropriate medication are developed (according to guidelines). Perioperatively, the prevalence of delirium is also recorded, measured by daily delirium screening (I-CAM) and RASS (Richmond Agitation Sedation Scale) for 7 days postoperatively, and then every 2 days until discharge or transfer from the index hospital if delirium persists. The duration and extent of restraints and sitting guards for hypermotor delirium with self-harm will also be recorded.

WP 3: Post-inpatient POCD assessment and transsectoral care.

Survey of primary and secondary endpoints: 1) prevalence of delirium measured by the Confusion Assessment Method (I-CAM), digit span backwards, and clinical assessment; and 2) persistent cognitive impairment 2 and 6 months after hospital stay measured by a neuropsychological test battery (MoCA, Trail-making Test A/B) and cognitive skills measured by continuous unstandardized test scores of these scales. In addition, trans-sectoral care will be improved with the following tools for primary care physicians: development of structured information for primary care physicians by the study team on the physical and cognitive situation, delirium history, any need for specialist co-treatment in case of delirium, suspected dementia or depression, medication recommendation, determination of rehabilitation requirements, scheduling of follow-up appointments, information transfer of knowledge of relatives, nursing service and primary care physician information through standardized transfer forms.

WP 4: Training courses

A total of three training curricula based on the previous knowledge and experience of the Stuttgart working group will be conducted in coordination with and with the collaboration of the other centers (see Table 2).

At the beginning of the study, the study teams of the participating hospitals will be trained in a **delirium master class** (up to 40 hours) in delirium screening, delirium syndrome and trigger diagnostics, delirium risk factors, dementias, as well as neuropsychological diagnostics, geriatric and psychiatric assessment according to a jointly responsible curriculum, in order to achieve a high degree of comparability. Before the start of the intervention, a structured introduction of the entire treatment team takes place on 3 different levels. For this purpose, the experience from the Bosch Foundation-funded projects (http://www.bosch-stiftung.de/content/language1/html/62538.asp 1 funding round Evang. Krankenhaus Bielefeld and 2. funding round Klinikum Stuttgart) is utilized. **A basic training** (90 min.) is to take place for all employees, from the cleaning staff to the head physician, **Delirium Scouts** are to maintain the daily ward work and **Delirium Champions** are to ensure the supervision and sustainable

implementation of the acquired knowledge**. Knowledge surveys before and after the training sessions as well as during the course are planned for process analysis.**

The preventive teams will be trained according to detailed manuals, and a corresponding manual will be created for the OR companionship module. They will be instructed and supervised by a specially trained psychogeriatric specialist who will individually "prescribe" and document the individual preventive modules, carry out delirium screenings and monitor standard compliance (e.g. pain, according to LL geriatric traumatology). The scope of duties of the approx. 10 delirium attendants includes the pre-, intra- and post-operative care and support of approx. 150 (planned) study patients per center as well as their care during their further inpatient stay.

WP 5: Multimodal delirium prevention/management

Non-pharmacological preventive measures are already implemented in the pre-hospital setting by means of educational talks and written recommendations as well as contact with the general practitioner for medication optimization (see WP 1).

Training program	Hrs.	Participant<br>Departments	Doctors	Nurses	Contents	Period implementation	Fields of activity	Work packages
**Delirium Master Class**								
	40	GZ<br>Anesthetists<br>Surgeons of different specialties, i.e.<br>Heart/ vascular	2<br>1<br>1<br>1	4<br>1	Delirium, screening, risk factors, cognitive deficits, neuropsychological tests, age-appropriate medicine, delirium complications, LL geriatric traumatology, POCD, dementia, diagnostics	Month 2	Recruitment, screening examination, delirium assessment, delirium prevention, geriatric and post-stat. assessments	WP 1<br>WP 2<br>WP 4<br>WP 5<br>WP 3
**Team-training**								
Level 1<br>– Basic training	1.5	Anesthetists, Surgeons, OR staff,	all	all	Delirium recognition, complications, commun-ication, prevention options	1 – 4 weeks before the start of the intervention	Applying knowledge of delirium, dealing with cognitive deficits	WP 5
Level 2<br>– Delirium Scout	10	Anesthetists, OR staff<br>Orthopedic and other Surgeons,<br>Heart/ vascular, OR staff	approx. 30<br>approx. 30<br>approx. 15	approx. 20<br>approx. 16<br>approx. 10	Delirium screening, NuDesc, complication avoidance, validation, prevention in everyday life, medication		Delirium screening, de-escalation, prevention	WP 2<br>WP 5
Level 3<br>– Delirium Champion	30	Anesthetists, OR staff<br>Orthopedic and other Surgeons,<br>Heart/vascular, OR staff	approx. 2<br>approx. 3<br>approx. 1	approx. 5/ 10<br>approx. 6<br>approx. 4	Delirium screening, I-CAM, causes, dementia, environ-mental design, guideline geriatric traumatology, restraints avoidance, delirium therapy		Delirium screening, cause finding, complication prevention, delirium therapy	WP2<br>WP 5
**Intervention Training**								

–Psychogeriatric specialist<br><br>(Delirium Champion)	+20	GZ, anesthetists, surgeons (orthopedics, heart/vascular)	2	3<br><br>1	Delirium risks, Delirium prevention modules, standards, LL geriatric traumatology, cognitive testing, observation in gerontological psychiatry 4 h	4 - 6 weeks before the start of the intervention	Arrangement & Supervision, Prevention modules, cog. testing, delirium screening I-CAM	WP 2<br><br>WP 5
– Prevention team<br><br>(Delirium Scout)	+30	GZ, anesthetists, surgeons (orthopedics, heart/vascular)		4	Training in prevention, activation, operation support, mobilization, sleep support, observation in geriatric psychiatry (4 h)		Implementation of surgery supervision, preventive modules, documentation	WP 5
**Trans sectoral training**								
– Basic instruction	0.75	General practitioners/ specialist<br><br>MFA	Lecture offer per centre		Delirium risk factors, delirium consequences, POCD, dementia, age-appropriate medication	at the start of the intervention	Medication adjustment pre-stat., support prevention	WP 4<br><br>WP 5
– POCD-Assessment	6	Memory Clinic,	1 doctor, 1 nurse per centre, MFA if necessary		Cognition testing, dementia diagnostics, scales	2 months after intervention	Post-stat. assessment, dementia detection	WP 3

Table 2: Training schedule with calculated staff requirements per center. Team training is offered 12 times per center in level 1, 7 times per center in level 2, and 3 times per center in level 3.

On the pilot wards, various multimodal modules will be introduced specifically for the high-risk patient group, supported by nursing, therapeutic and medical ward staff who will receive the training specified under WP 4 for this purpose.

In addition, **environmental measures** are implemented on according individual needs. The sensory organs, which generally deteriorate in old age, often lead to stress symptoms such as restlessness and anxiety, especially in dementia patients due to their limited perception processing. In order to adapt the hospital setting to the needs of cognitively impaired patients and to reduce complications typical of the clientele, such as falls, it is imperative to provide a structured environment that conveys security (e.g. signposting of the ward and the bathrooms with symbols, dementia-sensitive color design, bright lighting, night-time quietness protection, temporal orientation aids such as white boards, installation of analog clocks, appropriate aids for fall prevention (slipper socks) and orientation (signs etc.), participation support box for glasses, hearing aids, dentures, etc.).

Immediately after randomization, perioperative multimodal management, modified according to best practice models such as HELP (Inouye, Bogardus et al. 2000) and "The elderly in the operating room" (Gurlit and Mollmann 2008), will be implemented in all participating wards for 10-12 hours per day, 7 days per week. For this purpose, diagnostic and surgical accompaniment is provided by trained daily companions, nursing assistants, or volunteers from the time of inpatient admission. The psychogeriatric specialist (physician (delirium master), specialist nurse) of the respective departments monitor the implementation of the agreed treatment standards for perioperative management of patients at risk for delirium (premedication, surgery and anesthesia adjustment, pain monitoring, sufficient age-appropriate pain treatment, avoidance of movement restrictions (catheters, infusions, fixation), avoidance of benzodiazepines, pipamperone, melperone). Parallel to this, the psychogeriatric specialist carries out daily needs assessments of the individual measures required for delirium prevention for orientation, activation, movement promotion, meal support, anxiety relief, pain

monitoring, sleep disturbance (Bringemeier, Thomas et al. 2015). In addition, relatives are informed and trained to support and implement these measures.

WP 6: Data management

All data, including baseline data, will be collected on-site by study physicians and study nurses using eCRF and stored in web-based electronic data capture. No data will be extracted across clinics from databases such as ISH-Med for these assessments.

Work package 7 Evaluation (responsible M. Rapp):

Primary and secondary outcomes for the intervention are delirium prevalence, prevalence of POCD, multivariate change in cognitive skills in the postoperative course (MoCA, TMT A/B, digit span, FIM). The intervention study will be a stepped-wedge cluster randomized trial design. The primary outcome measure is the prevalence of delirium as measured by the Confusion Assessment Method (I-CAM), digit span backwards, and clinical assessment. Secondary outcomes are persistent cognitive impairment 2 and 6 months after hospital stay measured by a neuropsychological test battery (MoCA, Trail-making Test A/B) and cognitive skills measured by continuous unstandardized test scores of these scales. A cognitive deficit is defined here as the presence of a test score of < -0.5 standard deviations, normalized for age, gender, and education level, in one of these test procedures.

The case number estimate for the primary outcome (prevalence of delirium) assumes a reduction in the prevalence of delirium from 25% to 15% (Hshieh, Yue et al. 2015) as a result of the trans sectoral multimodality intervention. Here, a conventional analysis using Fisher's exact test yields a total case number of 514 patients with 1:1 randomization, given a power of $1-\beta = 0.80$, and an α-error of 5%. Using the adjustment formula proposed by Woertmann (Woertman, de Hoop et al. 2013) with 5 crossing points in a stepped-wedge design with a maximum of 25 patients per cluster per period and an intracluster correlation of 0.01, the correction factor for the stepped-wedge design is KF = 2.52, and thus the number of cases is 514 x 2.52 = 1295 patients. Assuming a dropout rate of 15%, this results in a case number of approximately 750 patients per arm. The minimum number of clusters is given by the ratio of the total number of patients to the product between the number of crossing points and the number of patients per cluster per period, and is 9.

For the secondary outcome of a reduction of persistent cognitive deficit from 20% to 10%, conventional analysis using Fisher's exact test yields a total number of 428 patients with 1:1 randomization, a power of $1-\beta = 0.80$, and an α-error of 5%, i.e., 1079 patients with design correction. For the primary outcome, logistic regression analyses with cluster adjustment are planned. For the secondary outcomes, additional mixed linear and logistic regression models with fixed and random factors will be performed to estimate heterogeneity over clusters (cf. Rapp et al. 2013)(Rapp, Mell et al. 2013).

WP 8 Health Economics (Responsible J. Wasem):

In a preparatory **module 8.1**, the data protection concept for health insurance data and the health economic evaluation concept are operationalized and validated. Milestone after 3 months: Data security and evaluation concept are developed.

In **module 8.2**, micro-costing is performed on a sample of 2 hospitals (Tübingen and Stuttgart) for all patients included there (using data from hospital administrations) to test the hypothesis that patients under the intervention incur lower costs of initial hospitalization for the hospitals. The respective §21 data set plus cost data is used for the study patients to determine the costs of the hospital stays in the groups of cases and controls. The aim is to determine the initial costs per delirium from the perspective of the service provider (hospital). Milestone after 12 months: Micro-costing performed.

In **module 8.3**, the expenses of the initial hospital stay are determined in a difference-in-difference approach from the perspective of the SHI (AOK Baden-Württemberg) for the patients insured by a specific health insurance (AOK) and compared to the outcome difference (delirium cases) to determine the cost-effectiveness. The target parameters are the costs per avoided delirium from the perspective of the health and long-term care insurer (AOK).

In **module 8.4**, a difference-in-difference approach from the perspective of the SHI (AOK Baden-Württemberg) is used to determine the expenditures in the 12 months before and after the index surgery for the AOK-insured and to compare them with the outcome difference (POCD cases) to determine the cost-effectiveness. The target parameters are the costs per avoided POCD case from the perspective of the health and long-term care insurer (AOK).

For modules 8.3 and 8.4, health insurance and long-term care insurance expenditures as well as utilization parameters, especially from the areas of hospital care, outpatient medical care, pharmaceutical care, the provision of remedies and aids, rehabilitation and long-term care, are considered. In work package 7, the AOK is informed - if necessary after data protection coordination via a data trust office - which of its patients (intervention patients and controls) are participating in the study (identification by means of health insurance number). At the same time, a pseudonym is assigned and communicated to the AOK. The AOK then transmits the pseudonymized health insurance data of the corresponding insured persons to the Chair of Medical Management. In addition, the data collected in WP 7 (especially delirium risk, delirium and POCD cases) can be pseudonymously transmitted to the Chair of Medical Management, where they can finally be linked to the routine data. To determine the cost-effectiveness, the costs in the intervention and control groups are compared and the incremental cost-effectiveness is calculated. Based on quality of life assessed by EQ-5D, quality-adjusted life years (QALYs) can be determined and used to calculate incremental cost-effectiveness. Where possible, subgroup analyses are performed (e.g., by age, sex, delirium risk) to examine whether cost-effectiveness differs across the relevant groups. Sensitivity analyses will also be performed. The analysis is performed according to the recommendations Good Practice Secondary Data Analysis (GPS).

WP 9 Consortium networking

The GZ Tübingen, with the entire UKT, carries out the consortial leadership. The 5 participating centers coordinate the processes in cooperation with surgery, anesthesia, and nursing management on site. Personnel resources are requested in the form of full staff positions for the coordination, implementation, and execution of the multimodal assessments at 4 outpatient and 2 inpatient time points. Funding is requested for multimodal intervention training and implementation in the 10 departments. Over three years, positions for physicians, nurses, and psychologists or nurse scientists are needed, as well as positions for volunteers, students, and senior nursing assistants for the interventions. Material costs are incurred for assessments and training. The data management is carried out by means of a secure online database to be financed under the leadership of Prof. Rapp. Costs for the health economic calculations by Prof Wasem based on the data of the AOK Baden-

Württemberg and the participating costing clinics are requested. For the data processing of the cost data by the AOK Baden-Württemberg, an expense allowance will be paid to the insurance company.

An external study-independent monitoring board with national experts will be established (Prof. Dr. Hewer, Göppingen, Prof. Dr. Gutzmann). The internal ethics board is under the leadership of PD Dr. M. Synofzik (Neurology Tübingen).

Work, time and milestone planning

Milestones (MS) listed in quarters (Chart 2):

Quartal	N	Q1	Q2	Q3	Q4	Q5	Q6	Q7	Q8	Q9	Q10	Q10		Q11	Q12
Dep 1	150	Preparation	Pre. Ass	Pre. Ass	Intervention	Intervention	Intervention	Intervention	Intervention	Post. Ass	Post. Ass	Post. Ass	P	12M Ass	12M M analysis
Dep 2	150	Preparation	Pre. Ass	Pre. Ass	Intervention	Intervention	Intervention	Intervention	Intervention	Post. Ass	Post. Ass	Post. Ass	r	12M Ass	12M M analysis
Dep 3	150	Preparation	Pre. Ass	Pre. Ass	Pre. Ass	Intervention	Intervention	Intervention	Intervention	Post. Ass	Post. Ass	Post. Ass	i	12M Ass	12M M analysis
Dep 4	150	Preparation	Pre. Ass	Pre. Ass	Pre. Ass	Intervention	Intervention	Intervention	Intervention	Post. Ass	Post. Ass	Post. Ass	m.	12M Ass	12M M analysis
Dep 5	150	Preparation	Pre. Ass	Pre. Ass	Pre. Ass	Pre. Ass	Intervention	Intervention	Intervention	Post. Ass	Post. Ass	Post. Ass		12M Ass	12M M analysis
Dep 6	150	Preparation	Pre. Ass	Pre. Ass	Pre. Ass	Pre. Ass	Intervention	Intervention	Intervention	Post. Ass	Post. Ass	Post. Ass	A	12M Ass	12M M analysis
Dep 7	150	Preparation	Pre. Ass	Pre. Ass	Pre. Ass	Pre. Ass	Pre. Ass	Intervention	Intervention	Post. Ass	Post. Ass	Post. Ass	n	12M Ass	12M M analysis
Dep 8	150	Preparation	Pre. Ass	Pre. Ass	Pre. Ass	Pre. Ass	Pre. Ass	Intervention	Intervention	Post. Ass	Post. Ass	Post. Ass	a	12M Ass	12M M analysis
Dep 9	150	Preparation	Pre. Ass	Pre. Ass	Pre. Ass	Pre. Ass	Pre. Ass	Pre. Ass	Intervention	Post. Ass	Post. Ass	Post. Ass	l.	12M Ass	12M M analysis
Dep 10	150	Preparation	Pre. Ass	Pre. Ass	Pre. Ass	Pre. Ass	Pre. Ass	Pre. Ass	Intervention	Post. Ass	Post. Ass	Post. Ass		12M Ass	12M M analysis
N	1500			250	250	250	250	250	250						

6 months MS 1: Contracts and preparations completed, in 300 pat. pre-stat. ass., 250 pat. peri-operative ass.
12 months MS 2: 2 dept. with 500 staff trained, 900 pat. Pre-stat. ass. 750 pat. perioperative ass.
18 months MS 3: 8 dept. with 2000 staff trained, 1500 pat. Pre-stat. ass., 1250 pat. periop., 640 pat. 6-month ass.
24 months MS 4: in 10 dept. 2500 staff trained, 1500 pat. periop., 1060 pat. 6 mo.-ass, 600 pat. 12 mo.-ass.
30 months MS 5: Evaluation delirium prevention stat., 1800 pat. evaluation delirium risk score, 1300 pat., 6 mo.-ass., 950 pat. 12 mo.-ass.
36 months MS 6: 1150 pat. 12 mo. ass., evaluation POCD prev. 6+12 mo., evaluation economics, publications.

7 Utilization Potential / Exploitation Potential

Successful implementation of our multicenter intervention studies will result in numerous practical implications for German health care practice:

1. Implementation of a clinically meaningful delirium risk score for estimating the delirium risk of older vulnerable people in prehospital care in Germany. In the future, the score should be collected in the prehospital setting by the general practitioner or in surgical or anesthesiologic outpatient clinics by geriatrics-trained physicians and specialized nurses (geriatrics, geriatric psychiatry) and should include appropriate medication management. This will enable the efficient use of the intervention in patients at risk.

2. Collect valid delirium incidences and POCD incidences at 6 months and 12 months for the most common elective surgeries. These could serve as benchmarks for quality of care for surgical departments in patients at risk for delirium/ POCD. The severity of the procedures and the frailty of the patients will be taken into account in the score (heart, thoracic and vascular surgery, orthopedics and other surgery).

3. Evaluation of a new, cross-sectoral, multimodal delirium and POCD prevention intervention with high effectiveness in actual German health care realities. The comprehensive team training and

interventions will permeate the departments and thus can be applied to other, non-elective, patients and procedures.

4. Cost-efficiency calculation of the preventive effort and the economic long-term effect, taking into account the training costs, intervention costs, savings through complication avoidance and shortening of the length of stay as well as long-term reduction of care costs will take place for the first time in Germany. For the participating patients of the AOK Baden-Württemberg (approx. 50% of the total), trans-sectoral costs for physician visits, medications, rehabilitation and nursing measures up to month 12 postoperatively are recorded and compared to the avoided POCD cases. For the first time, the costs and savings of the initial hospitalization in this risk group are also determined from the perspective of the hospitals.

8 Ethical/legal aspects

This is an intervention study in which existing guidelines and recommendations of Professional Scientific organizations (including recommendations of the Working group "Orthogeriatric trauma surgery" of the German Traumatology Association, NICE guidelines, AGS guidelines) are to be implemented. Therefore, it is not a study according to the AMG/MPG law (laws regarding medication and medical products). An ethics vote according to the Professional Code of Conduct will be obtained for all centers. In the pre-hospital assessments, it can be expected that about 40% of the examined elderly people might have mild or severe cognitive impairment (GhoSt study 2016, Bickel et al). The patients and, if applicable, their relatives must be made aware that the examinations could give rise to the suspicion of dementia and that this can have consequences for the self-image of the person concerned (Klöppel et al, 2016). If a mild cognitive disorder or incipient dementia is disclosed in the screening, the ability to consent and to contract is not yet restricted. According to Vollmann, consent ability does exist until the transition to more severe dementia (MMST < 15 P., MoCA < 8 P.) In participating patients, the benefit outweighs the harms, as the intervention should reduce the risk of delirium and POD. A possible harm would be the self-stigmatization as cognitively impaired and at risk for dementia.

In the case of a previously unrecognized severe cognitive disorder of the extent of moderate or severe dementia (if these symptoms already restrict the daily function for 6 months), the ability to consent to the intervention would probably not be preserved. In case of an existing substitute decision-maker, the authorized representative or legal guardian would have to be informed about the participation in the study and about the elective surgical procedure and would have to decide accordingly in the patient's best interest.

<u>Discontinuation criteria for the individual subject</u>: Withdrawal by patient or caregiver/legal guardian or death.

<u>Discontinuation criteria for the intervention</u>: SAEs are recorded and evaluated at quarterly intervals. Falls, strokes, infections, and other serious perioperative complications are expected in the patient group independent of intervention.

Ethics board of the overall study (EBG): An external study-independent monitoring board with national experts is established (Prof. Hewer, Prof. Gutzmann). The internal ethics board will be chaired by PD Dr. M. Synofzik (Neurological Clinic and GZ Tübingen).

Data protection: A commercially available database with pseudonymized data will be created. A 128 bit SSL connection will be used to encrypt the data during transmission over the internet.

9 Risk factors

Quality assurance and feasibility of the project during the duration of funding

The participating hospitals have been working together in the State Geriatrics Working Group for 20 years and have large surgical departments (>1000 procedures/year each). Their medical directors -as well as the hospital management, anesthesia, and nursing management- document their interest in the introduction and evaluation of delirium prevention measures and their willingness to recruit the necessary patients as well as to cooperate in the study to the extent requested. Nursing directors and anesthesia departments, which also operate the intensive care units, have confirmed their cooperation. The work, time and milestone plan is therefore realistic. In Tübingen, for example, 270 surgical procedures were performed on the heart in cardiac and vascular surgery and in orthopedics almost 400 (184 operations with joint replacement (S-82) and 200 on the spine) in patients over 70 years of age in 2015.

In the outpatient sector, there is a close connection to the referring specialist practices (e.g. orthopedics, cardiology, angiology/vascular diseases) and to general practitioners, including the geriatric centers. At the Department of General Medicine, UKT, there is a cooperation with general practitioners, who usually do the postoperative management and who might invite the post-inpatient assessments to their offices.

Recruitment

If a department is unable to meet the recruitment target of 25 patients in the first quarter (Q3), concrete and binding measures will be initiated to reach the patient number. Here, each center makes a binding commitment to either increase recruitment in the department or include another surgical department in the training sessions (e.g., 2nd Orthopedics, Urology, etc.). If multiple departments do not meet their target, the 5 recruitment blocks would be extended from 3 months in length each to 4 months, thus 33% more time would be given to recruit. The POCD assessments would accordingly be completed after 6 months instead of 12 months for the 2nd half of the patients to be recruited (from Q6).

10 Financing plan

The financing plan mainly includes the costs of personnel for the implementation of the 9 work packages at the 6 sites, in total 912 person months (40h/week, E9 and E14 salary positions):

**10.1**	**Total personnel expenses (for itemized list, see financial plan)**	4.908.000
**10.2**	**Non-personnel expenses**	**€**
10.2.1-3	Material costs and travel	95.000
10.2.4	Infrastructure allowance (up to 25% of personnel expenses, see 7.1)	1.227.000
**10.2**	**Total non-personnel expenses**	**1.322.000**
**10.3**	**Investments (individual price > 410 € net)**	**€**
	Secutrial database	30.000
**10.3**	**Total investments**	**30.000**
**10.4**	**Total expenditure of the project**	**€**
10.4.1	Sum of personnel expenses	4.908.00

10.4.2	Sum of non-personnel expenses	1.322.000
10.4.3	Sum of investments	30.000
**10.4**	**Total expenditure of the project**	**6.260.000**

11 Signatures

Aug 16th, 2016 Head of Consortium **Prof. Dr. Gerhard Eschweiler**

Responsible for the Methodology **Prof. Dr. Michael Rapp**

12 References

American Geriatrics Society Expert Panel on Postoperative Delirium in Older Adults (2015). "Postoperative delirium in older adults: best practice statement from the American Geriatrics Society." J Am Coll Surg **220**(2): 136-148 e131.

Bakker, F. C., A. Persoon, S. J. Bredie, J. van Haren-Willems, V. J. Leferink, L. Noyez, Y. Schoon and M. G. Olde Rikkert (2014). "The CareWell in Hospital program to improve the quality of care for frail elderly inpatients: results of a before-after study with focus on surgical patients." Am J Surg **208**(5): 735-746.

Bringemeier, J., C. Thomas, M. Guhra and S. Kreisel (2015). "HELP zur Vermeidung von Delirien." PSYCH up2date **9**(03): 137-148.

Brown, C. H. t., C. Morrissey, M. Ono, G. Yenokyan, O. A. Selnes, J. Walston, L. Max, A. LaFlam, K. Neufeld, R. F. Gottesman and C. W. Hogue (2015). "Impaired olfaction and risk of delirium or cognitive decline after cardiac surgery." J Am Geriatr Soc **63**(1): 16-23.

Charlson, M. E., P. Pompei, K. L. Ales and C. R. MacKenzie (1987). "A new method of classifying prognostic comorbidity in longitudinal studies: development and validation." J Chron Dis **40**(5): 373-383.

Fong, T. G., D. Davis, M. E. Growdon, A. Albuquerque and S. K. Inouye (2015). "The interface between delirium and dementia in elderly adults." Lancet Neurol **14**(8): 823-832.

Fong, T. G., R. N. Jones, P. Shi, E. R. Marcantonio, L. Yap, J. L. Rudolph, F. M. Yang, D. K. Kiely and S. K. Inouye (2009). "Delirium accelerates cognitive decline in Alzheimer disease." Neurology **72**(18): 1570-1575.

Gleason, L. J., E. M. Schmitt, C. M. Kosar, P. Tabloski, J. S. Saczynski, T. Robinson, Z. Cooper, S. O. Rogers, Jr., R. N. Jones, E. R. Marcantonio and S. K. Inouye (2015). "Effect of Delirium and Other Major Complications on Outcomes After Elective Surgery in Older Adults." JAMA Surg **150**(12): 1134-1140.

Guenther, U., L. Riedel and F. M. Radtke (2016). "Patients prone for postoperative delirium: preoperative assessment, perioperative prophylaxis, postoperative treatment." Curr Opin Anaesthesiol. 2016 Jun;**29**(3):384-90.

Klöppel S, Peter J, Ludl A, Pilatus A, Maier S, Mader I, Heimbach B, Frings L, Egger K, Dukart J, Schroeter ML, Perneczky R, Häussermann P, Vach W, rbach H,Teipel S, Hüll M, Abdulkadir A. Applying

Automated MR-Based Diagnostic Methods to the Memory Clinic: A Prospective Study. J Alzheimers Dis. 2015 Aug 11;**47**(4):939-54.

Gurlit, S. and M. Mollmann (2008). "How to prevent perioperative delirium in the elderly?" Z Gerontol Geriatr **41**(6): 447-452; http://www.mgepa.nrw.de/mediapool/pdf/presse/pressemitteilungen/Der_alte_Mensch_im_OP.pdf .

Hagen, K., A. C. Ehlis, F. B. Haeussinger, S. Beeretz, G. V. Kromer, S. Heinzel, W. Maetzler, G. W. Eschweiler, D. Berg, A. J. Fallgatter, F. G. Metzger and T. S. Consortium (2015). "The relation of SMI and the VSEP in a risk sample for neurodegenerative disorders." J Neural Transm (Vienna) **122**(8): 1167-1174.

Hessler, J. B., M. Bronner, T. Etgen, O. Gotzler, H. Forstl, H. Poppert, D. Sander and H. Bickel (2015). "Smoking increases the risk of delirium for older inpatients: a prospective population-based study." Gen Hosp Psychiatry **37**(4): 360-364.

Hshieh, T. T., J. Yue, E. Oh, M. Puelle, S. Dowal, T. Travison and S. K. Inouye (2015). "Effectiveness of multicomponent nonpharmacological delirium interventions: a meta-analysis." JAMA Intern Med **175**(4): 512-520.

Inouye, S. K. (2000). "Prevention of delirium in hospitalized older patients: risk factors and targeted intervention strategies." Ann Med **32**(4): 257-263.

Inouye, S. K. (2006). "Delirium in older persons." N Engl J Med **354**(11): 1157-1165.

Inouye, S. K., S. T. Bogardus, Jr., D. I. Baker, L. Leo-Summers and L. M. Cooney, Jr. (2000). "The Hospital Elder Life Program: a model of care to prevent cognitive and functional decline in older hospitalized patients. Hospital Elder Life Program." J Am Geriatr Soc **48**(12): 1697-1706.

Jessen, F., S. Wolfsgruber, B. Wiese, H. Bickel, E. Mosch, H. Kaduszkiewicz, M. Pentzek, S. G. Riedel-Heller, T. Luck, A. Fuchs, S. Weyerer, J. Werle, H. van den Bussche, M. Scherer, W. Maier, M. Wagner, C. German Study on Aging and P. Dementia in Primary Care (2014). "AD dementia risk in late MCI, in early MCI, and in subjective memory impairment." Alzheimers Dement **10**(1): 76-83.

Jorm, A. F. (1994). "A short form of the Informant Questionnaire on Cognitive Decline in the Elderly (IQCODE): development and cross-validation." Psychol Med **24**(1): 145-153.

Monk, T. G. and C. C. Price (2011). "Postoperative cognitive disorders." Curr Opin Crit Care **17**(4): 376-381.

Oh, E. S., M. Li, T. M. Fafowora, S. K. Inouye, C. H. Chen, L. M. Rosman, C. G. Lyketsos, F. E. Sieber and M. A. Puhan (2015). "Preoperative risk factors for postoperative delirium following hip fracture repair: a systematic review." Int J Geriatr Psychiatry **30**(9): 900-910.

Patel, J., J. Baldwin, P. Bunting and S. Laha (2014). "The effect of a multicomponent multidisciplinary bundle of interventions on sleep and delirium in medical and surgical intensive care patients." Anaesthesia **69**(6): 540-549.

Pretto, M., R. Spirig, K. Milisen, S. Degeest, P. Regazzoni and W. Hasemann (2009). "Effects of an interdisciplinary nurse-led Delirium Prevention and Management Program (DPMP) on nursing workload: a pilot study." Int J Nurs Stud **46**(6): 804-812.

Rapp, M. A., T. Mell, T. Majic, Y. Treusch, J. Nordheim, M. Niemann-Mirmehdi, H. Gutzmann and A. Heinz (2013). "Agitation in nursing home residents with dementia (VIDEANT trial): effects of a cluster-randomized, controlled, guideline implementation trial." J Am Med Dir Assoc **14**(9): 690-695.

Reston, J. T. and K. M. Schoelles (2013). "In-facility delirium prevention programs as a patient safety strategy: a systematic review." Ann Intern Med **158**(5 Pt 2): 375-380.

Rudolph, J. L., S. K. Inouye, R. N. Jones, F. M. Yang, T. G. Fong, S. E. Levkoff and E. R. Marcantonio (2010). "Delirium: an independent predictor of functional decline after cardiac surgery." J Am Geriatr Soc **58**(4): 643-649.

Rudolph, J. L., R. N. Jones, L. S. Rasmussen, J. H. Silverstein, S. K. Inouye and E. R. Marcantonio (2007). "Independent vascular and cognitive risk factors for postoperative delirium." Am J Med **120**(9): 807-813.

Rundshagen, I. (2014). "Postoperative cognitive dysfunction." Dtsch Arztebl Int **111**(8): 119-125.

Siddiqi, N., J. K. Harrison, A. Clegg, E. A. Teale, J. Young, J. Taylor and S. A. Simpkins (2016). "Interventions for preventing delirium in hospitalised non-ICU patients." Cochrane Database Syst Rev **3**: CD005563.

Thomas, C., S. H. Kreisel, P. Oster, M. Driessen, V. Arolt and S. K. Inouye (2012). "Diagnosing delirium in older hospitalized adults with dementia: adapting the confusion assessment method to international classification of diseases, tenth revision, diagnostic criteria." J Am Geriatr Soc **60**(8): 1471-1477.

Todd, O., L. Gellrich, A. Maclullich, M. Driessen, C. Thomas and S. Kreisel "Sleep deficiency is an independent risk factor for post-operative delirium in older people undergoing elective surgery: a prospective cohort study." JAGS: (under revision).

Visser, L., A. Prent, M. J. van der Laan, B. L. van Leeuwen, G. J. Izaks, C. J. Zeebregts and R. A. Pol (2015). "Predicting postoperative delirium after vascular surgical procedures." J Vasc Surg **62**(1): 183-189.

Wehling, M., H. Burkhardt, A. Kuhn-Thiel, F. Pazan, C. Throm, C. Weiss and H. Frohnhofen (2016). "VALFORTA: a randomised trial to validate the FORTA (Fit fOR The Aged) classification." Age Ageing **45**(2): 262-267.

Witlox, J., L. S. Eurelings, J. F. de Jonghe, K. J. Kalisvaart, P. Eikelenboom and W. A. van Gool (2010). "Delirium in elderly patients and the risk of postdischarge mortality, institutionalization, and dementia: a meta-analysis." JAMA **304**(4): 443-451.

Woertman, W., E. de Hoop, M. Moerbeek, S. U. Zuidema, D. L. Gerritsen and S. Teerenstra (2013). "Stepped wedge designs could reduce the required sample size in cluster randomized trials." J Clin Epidemiol **66**(7): 752-758.

Yang FM, Inouye SK, Fearing MA, Kiely DK, Marcantonio ER, Jones RN. “Participation in activity and risk for incident delirium.” J Am Geriatr Soc, 2008 Aug;**56**(8):1479-84.

Zhang H, Lu Y, Liu M, Zou Z, Wang L, Xu FY, Shi XY. “Strategies for prevention of postoperative delirium: a systematic review and meta-analysis of randomized trials.” Crit Care, 2013 Mar 18;**17**(2): R47.
