# Supplementary material for: Outcomes of a Delirium Prevention Program in Older Persons After Elective Surgery: A Stepped-Wedge Cluster Randomized Clinical Trial
Source: JAMA Surg. 2021 Dec 15;157(2):e216370. doi: 10.1001/jamasurg.2021.6370 (PMC8674802; doi:10.1001/jamasurg.2021.6370)
Supplement: Supplement 2. — eTable 1. Baseline characteristics of participants in each centre. eTable 2. List of surgical procedures performed. eTable 3. Missing data for the main study variables. eTable 4. Delirium rate (%) per period in each centre. eTable 5. GEE analysis (model 2). eTable 6. GEE analysis (model 3) of cardiac and noncardiac surgery group. eTable 7. Effect of the intervention on overall delirium duration. [file jamasurg-e216370-s002.pdf]

Supplemental Online Content

Deeken F, Sánchez A, Rapp MA, et al; PAWEL Study Group. Outcomes of a delirium prevention program in older persons after elective surgery: a stepped-wedge cluster randomized clinical trial. *JAMA Surg*. Published online December 15, 2021. doi:10.1001/jamasurg.2021.6370

eTable 1. Baseline characteristics of participants in each centre
eTable 2. List of surgical procedures performed
eTable 3. Missing data for the main study variables
eTable 4. Delirium rate (%) per period in each centre
eTable 5. GEE analysis (model 2)
eTable 6. GEE analysis (model 3) of cardiac and noncardiac surgery group
eTable 7. Effect of the intervention on overall delirium duration

This supplementary material has been provided by the authors to give readers additional information about their work.

© 2021 Deeken F et al. *JAMA Surgery*.

eTable 1. Baseline characteristics of participants in each centre

	No./Total No. (%)									
	Centre 1		Centre 2		Centre 3		Centre 4		Centre 5	
**Characteristic**	**IG (n = 239)[a]**	**CG (n = 45)[a]**	**IG (n = 200)[a]**	**CG (n = 102)[a]**	**IG (n = 149)[a]**	**CG (n = 145)[a]**	**IG (n = 105)[a]**	**CG (n = 188)[a]**	**IG (n = 47)[a]**	**CG (n = 250)[a]**
Age (years), median (IQR), min-max	77 (74-81), 70-90	78 (73-81), 71-93	77 (74-80), 70-93	76 (73-78), 70-90	78 (75-82), 70-98	79 (76-81), 70-95	79 (75-83), 70-90	79 (75-82), 70-96	77 (75-80), 70-86	78 (74-80), 70-93
Male	144/239 (60.3)	23/45 (51.1)	112/200 (56.0)	56/102 (54.9)	62/149 (41.6)	70/145 (48.3)	57/105 (54.3)	77/188 (41.0)	28/47 (59.6)	134/250 (53.6)
Education (years), median (IQR)	13 (12-17)	13 (12-17)	12 (12-13)	12 (12-13)	12 (12-17)	12 (11-13)	12 (12-14)	12 (9-13)	12 (12-17)	12 (11-13)
MoCA score, median (IQR)	24 (21-26)	24 (21-27)	24 (21-25)	24 (21-26)	23 (19-25)	23 (19-26)	24 (21-26)	24 (22-26)	23 (20-25)	24 (22-26)
SMI	148/229 (64.6)	35/43 (81.4)	119/200 (59.5)	49/99 (49.5)	75/146 (51.4)	71/145 (49.0)	55/104 (52.9)	89/180 (49.4)	25/47 (53.2)	115/245 (46.9)
CCI score, median (IQR)	3 (1-5)	3 (1-4)	2 (1-3)	1 (1-2)	1 (1-3)	1 (1-2)	2 (1-4)	2 (1-3)	1 (1-3)	2 (1-3)
Dementia (CCI)	9/239 (3.8)	0/45 (0.0)	3/200 (1.5)	0/102 (0.0)	3/149 (2.0)	4/145 (2.8)	0/105 (0.0)	3/188 (1.6)	1/47 (2.1)	5/250 (2.0)
CSHA-CFS score, median (IQR)	3 (2-3)	3 (2-4)	3 (3-4)	3 (2-4)	4 (3-6)	4 (3-6)	3 (3-4)	3 (3-4)	3 (3-4)	4 (3-5)
Polypharmacy	130/223 (58.3)	27/43 (62.8)	129/196 (65.8)	60/99 (60.6)	99/141 (70.2)	104/140 (74.3)	67/100 (67.0)	104/183 (56.8)	28/47 (59.6)	186/244 (76.2)
Type of surgery										
Cardiac or vascular surgery	98/239 (41.0)	14/45 (31.1)	99/200 (49.5)	52/102 (51.0)	0/149 (0.0)	0/145 (0.0)	51/105 (48.6)	78/188 (41.5)	25/47 (53.2)	115/250 (46.0)
Orthopedic/spine surgery	60/239 (25.1)	15/45 (33.3)	101/200 (50.5)	50/102 (49.0)	117/149 (78.5)	114/145 (78.6)	54/105 (51.4)	110/188 (58.5)	20/47 (42.6)	101/250 (40.4)
Abdominal surgery	46/239 (19.2)	12/45 (26.7)	0/200 (0.0)	0/102 (0.0)	27/149 (18.1)	30/145 (20.7)	0/105 (0.0)	0/188 (0.0)	2/47 (4.3)	26/250 (10.4)
Other surgery	35/239 (14.6)	4/45 (8.9)	0/200 (0.0)	0/102 (0.0)	5/149 (3.4)	1/145 (0.7)	0/105 (0.0)	0/188 (0.0)	0/47 (0.0)	8/250 (3.2)

Abbreviations: CCI, Charlson Comorbidity Index; CG: control group; CSHA-CFS, Canadian Study of Health & Ageing- Clinical Frailty Scale; IG: intervention group; IQR, interquartile range; MoCA, Montreal Cognitive Assessment; SMI, subjective memory impairment.
[a] Unless otherwise stated.

© 2021 Deeken F et al. *JAMA Surgery*.

eTable 2. List of surgical procedures performed

Specialty	Type of Surgery	Surgical Procedures	Total Sample (N = 1470)
Cardiac (n = 379)	Valves	Replacement of aortic and/or mitral valve by prosthesis	57
	Multiple procedures	Prosthetic heart valve and coronary-artery bypass	52
		Heart valve prosthesis and rhythm surgery and others	26
		Valvuloplasty, partly including surgery for arrhythmia	48
		Minimally invasive heart valve surgery	29
	Coronary Artery Bypass	Bypass, single	23
		Bypass, double; some including rhythm surgery	52
		Triple bypass and more	43
		Aorto-coronary bypass, minimally invasive	35
	Conduction system	Pacemaker insertions, ablations and others	14
Vascular (n = 153)	Endarterectomy		
	Head	Carotid endarterectomy	48
	Lower body	Femoral and/or pelvic artery endarterectomy	25
	Aortic	Resection and interposition at the aorta	16
	Endovascular	Endovascular implantation of stent prostheses, aortic aneurysm repair	33
	Other	Arteriovenous shunt, iliac or femoral bypass, patchplasty	17
		Vascular surgery, not specified	14
Orthopedic /Spine (n = 742)	Spine (n = 99)		
	Cervical or thoracic	Laminectomy and spinal/root decompression	5
	Lumbar	Laminectomy/ hemilaminectomy and root decompression	13
		Spondylodesis	43
		Discectomy	10
	Minimally invasive	Discectomy and/ or spondylodesis	16
	Other	Vertebral body replacement, neoplasm, Spine surgery, not further specified (n = 9)	12

© 2021 Deeken F et al. *JAMA Surgery*.

	Joint Replacement (n = 575)<br>Hip	Implantation of a hip endoprosthesis	248
		Revision, replacement or removal of hip endoprosthesis	32
	Knee	Implantation of a knee endoprosthesis	219
		Revision, replacement, removal of knee endoprosthesis	24
	Upper extremity	Implantation of upper extremity joint endoprosthesis	40
		Revision, replacement, removal of upper extremity joint endoprosthesis	12
	Fractures and luxations	Open joint surgery	9
		Open reduction of a fracture	27
		Closed reduction of fracture, dislocation, mainly femur	13
	Other	Bone transposition, osteosynthesis material removal, misc.	19
Abdominal	Gastrointestinal (n = 73)	Esophagus surgery	9
		Gastrectomy, gastroenterostomy	14
		Partial resection of the colon	28
		Enterostomy, rectum and anus surgeries	22
		Operations on the hepatic, biliary or pancreatic system, splenectomy	32
	Herniae	abdominal hernia repair	21
Urogenital		Nephrectomy, total or partial	21
		Cystectomy, radical or partial, Prostatectomy, Ureter surgery, not further specified (n = 2)	35
Visceral and Other	Endocrine glands	Surgery on thyroid and parathyroid gland	5
		Adrenalectomy	2
	Lung	Pulmonary and bronchial surgery, lobectomy	3
	Other	Parotid surgery, other neoplasms, Lymphadenectomy	4

*According to the German ICD-10 Operation and Procedure Classification System (OPS), chapter 5 - operations, resembling the international ICD-10 Procedure Coding System (ICD-10-PCS).

© 2021 Deeken F et al. *JAMA Surgery*.

eTable 3. Missing data for the main study variables

Characteristic	Missing data (%)		
	**Total sample**	**IG**	**CG**
Delirium	0.0	0.0	0.0
Intervention	0.0	0.0	0.0
Age (years)	0.0	0.0	0.0
Male	0.0	0.0	0.0
Education (years)	0.3	0.4	0.1
MoCA score	0.8	0.9	0.7
SMI	2.2	1.9	2.5
CCI score	0.0	0.0	0.0
Dementia (CCI)	0.0	0.0	0.0
CSHA-CFS score	1.0	1.6	0.4
Polypharmacy	3.7	4.5	2.9
Type of surgery	0.0	0.0	0.0
Length of stay	0.2	0.4	0.0
Days with delirium	0.0	0.0	0.0
Percentage of days with delirium	0.1	0.1	0.0

Abbreviations: CCI, Charlson Comorbidity Index; CG, control group; CSHA, CSHA Clinical Frailty Scale; IG, intervention group; MoCA, Montreal Cognitive Assessment; SMI, subjective memory impairment.

© 2021 Deeken F et al. *JAMA Surgery*.

eTable 4. Delirium rate (%) per period in each centre

	n	**Interval 1** (20.11.2017-18.02.2018)	**Interval 2** (19.02.2018-13.05.2018)	**Interval 3** (14.05.2018-05.08.2018)	**Interval 4** (06.08.2018-28.10.2018)	**Interval 5** (29.10.2018-20.01.2019)	**Interval 6** (21.01.2019-12.04.2019)	**Total (%)**
**Centre 1**	284	15.6	17.4	7.7	16.0	6.5	15.6	13.0
**Centre 2**	302	34.0	34.7	26.5	44.2	21.3	13.8	32.1
**Centre 3**	294	13.5	20.0	6.3	11.1	9.8	9.4	11.6
**Centre 4**	293	14.3	28.9	12.2	23.1	13.8	17.0	21.2
**Centre 5**	297	38.8	38.3	18.4	25.5	26.0	31.9	29.6
**Total (%)**		22.7	28.0	14.2	24.4	19.1	20.8	

© 2021 Deeken F et al. *JAMA Surgery*.

eTable 5. GEE analysis (model 2)[a]

	Coefficient (B)	S.E	Wald X^2	OR (95% CI)	*P* value	RRR (95% CI)
Constant	-0.82	1.17	0.493	0.44 (0.05 to 4.34)	.48	83.6% (30.9 to 96.1)
Intervention (Intervention group)	-0.19	0.07	7.892	0.82 (0.72 to 0.94)	.005	13.0% (1.7 to 23.0)
Male	0.58	0.11	28.550	1.78 (1.44 to 2.21)	<.001	-48.7% (-82.3 to -21.3)
Education (years)	-0.01	0.03	0.154	0.99 (0.93 to 1.05)	.70	0.7% (-3.4 to 4.6)
Subjective Memory Impairment	0.09	0.15	0.362	1.10 (0.81 to 1.48)	.55	-7.1% (-31.1 to 12.4)
Comorbidity (CCI score)	0.01	0.04	0.116	1.01 (0.94 to 1.10)	.73	-1.0% (-5.8 to 3.6)
Frailty (CSHA-CFS score)	0.26	0.07	15.075	1.30 (1.14 to 1.49)	<.001	-18.1% (-25.2 to 11.3)
Type of surgery[b]						
Cardiac surgery	0.95	0.34	7.698	2.59 (1.32 to 5.04)	.006	-91.2% (-176.8 to -32.2)
Orthopedic/spine surgery	-0.69	0.34	4.083	0.50 (0.26 to 0.98)	.04	36.1% (0.5 to 58.9)
Abdominal surgery	-0.30	0.24	1.575	0.74 (0.47 to 1.18)	.21	23.3% (-0.4 to 41.5)
Age	0.03	0.01	4.998	1.03 (1.01 to 1.06)	.01	-2.1% (-4.0 to -0.3)
MoCA score	-0.12	0.02	28.186	0.89 (0.85 to 0.93)	<.001	7.3% (4.1 to 10.4)
Dementia	1.49	0.52	8.307	4.44 (1.61 to 12.28)	.004	36.1% (10.5 to 54.4)
Polypharmacy	0.06	0.10	0.365	0.97 (0.76 to 1.24)	.82	4.8% (-13.6 to 20.3)

Abbreviations: CCI, Charlson Comorbidity Index; CG, control group; CI, confidence interval; CSHA, CSHA Clinical Frailty Scale; GEE, Generalized Estimating Equations; IG, intervention group; MoCA, Montreal Cognitive Assessment; RRR, relative risk reduction; SMI, subjective memory impairment.
Quasi Likelihood Under Independence Model Criterion (QIC): 1262.874
Corrected QIC (QICC): 1227.139
[a] Adjusted for all variables that were significant between IG (n = 681) and CG (n =684) at baseline and major risk factors of delirium.
[b] Reference category: other surgery.

© 2021 Deeken F et al. *JAMA Surgery*.

eTable 6. GEE analysis (model 3) of cardiac and noncardiac surgery group

eTable 6a. GEE analysis (model 3). Cardiac surgery group[a]

	**Coefficient (B)**	**S.E**	**Wald X^2**	**Odds ratio (95% CI)**	***P* value**	**RRR (95% CI)**
Constant	-2.96	1.88	2.469	0.05 (0.00 to 2.08)	.12	94.2% (25.4 to 99.5)
Intervention (Intervention group)	0.16	0.27	0.373	1.18 (0.70 to 1.99)	.54	-10.6% (-52.5 to 19.9)
Male	0.24	0.08	9.752	1.27 (1.09 to 1.48)	.002	-16.0% (-28.6 to -4.6)
Education (years)	-0.01	0.03	0.108	0.99 (0.92 to 1.06)	.74	0.6% (-3.8 to 4.7)
Subjective Memory Impairment	0.07	0.25	0.065	1.07 (0.65 to 1.75)	.80	-4.2% (-41.1 to 23.0)
Comorbidity (CCI score)	0.06	0.03	3.152	1.06 (0.99 to 1.13)	.08	-3.6% (-6.6 to -0.7)
Frailty (CSHA-CFS score)	0.26	0.12	4.629	1.30 (1.02 to 1.65)	.03	-16.0% (-29.4 to -4.1)
Age	0.05	0.03	2.985	1.05 (0.99 to 1.12)	.08	-3.4% (-7.5 to 0.6)
MoCA score	-0.09	0.02	34.340	0.92 (0.89 to 0.94)	<.001	5.1% (2.9 to 7.2)
Dementia	0.65	0.33	3.763	1.92 (0.99 to 3.67)	.05	19.9% (-17.4 to 45.3)
Polypharmacy	0.06	0.28	0.042	1.06 (0.62 to 1.85)	.84	4.1% (-38.0 to 33.4)

Abbreviations: CCI, Charlson Comorbidity Index; CG, control group; CI, confidence interval; CSHA, CSHA Clinical Frailty Scale; GEE, Generalized Estimating Equations; IG, intervention group; MoCA, Montreal Cognitive Assessment; RRR, relative risk reduction; SMI, subjective memory impairment.

Quasi Likelihood Under Independence Model Criterion (QIC): 700.594

Corrected QICC (QICC): 664.642

[a] Adjusted for all variables that were significant between IG (n = 259) and CG (n = 249) at baseline and major risk factors of delirium.

© 2021 Deeken F et al. *JAMA Surgery*.

eTable 6b. GEE analysis (model 3). Non-cardiac surgery group[a]

	Coefficient (B)	S.E	Wald X^2	OR (95% CI)	*P* value	RRR (95% CI)
Constant	-2.43	1.51	2.583	0.09 (0.01 to 1.71)	.11	95.1% (72.0 to 99.1)
Intervention (Intervention group)	-0.54	0.27	3.944	0.59 (0.35 to 0.99)	.047	28.5% (-3.5 to 50.6)
Male	0.78	0.08	86.652	2.18 (1.85 to 2.57)	<.001	-78.6% (-104.1 to -56.2)
Education (years)	0.03	0.02	1.386	1.03 (0.98 to 1.08)	.24	-1.7% (-5.5 to 1.9)
Subjective Memory Impairment	0.13	0.20	0.416	1.14 (0.77 to 1.67)	.52	-11.8% (-51.3 to 17.3)
Comorbidity (CCI score)	0.04	0.04	0.736	1.04 (0.95 to 1.13)	.39	-2.1% (-6.5 to 2.1)
Frailty (CSHA-CFS score)	0.20	0.05	15.633	1.22 (1.10 to 1.34)	<.001	-12.7% (-19.5 to -6.4)
Age	0.05	0.02	9.717	1.05 (1.02 to 1.08)	.002	-3.7% (-5.6 to -1.9)
MoCA score	-0.15	0.03	28.660	0.86 (0.81 to 0.91)	<.001	10.1% (6.4 to 13.7)
Dementia	-1.65	0.53	9.565	5.21 (1.83 to 14.70)	.002	41.3% (18.9 to 57.5)
Polypharmacy	-0.10	0.20	0.259	0.90 (0.61 to 1.34)	.61	-7.8% (-45.1 to 19.9)

Abbreviations: CCI, Charlson Comorbidity Index; CG, control group; CI, confidence interval; CSHA, CSHA Clinical Frailty Scale; GEE, Generalized Estimating Equations; IG, intervention group; MoCA, Montreal Cognitive Assessment; RRR, relative risk reduction; SMI, subjective memory impairment. CCI, Charlson Comorbidity Index; CG, control group; CI, confidence interval; CSHA, CSHA Clinical Frailty Scale; GEE, Generalized Estimating Equations; IG, intervention group; MoCA, Montreal Cognitive Assessment; RRR, relative risk reduction; SMI, subjective memory impairment.

Quasi Likelihood Under Independence Model Criterion (QIC): 568.484

Corrected QIC (QICC): 570.148

[a] Adjusted for all variables that were significant between IG (n = 422) and CG (n = 435) at baseline and major risk factors of delirium.

eTable 7. Effect of the intervention on overall delirium duration

			Length of stay (days)		Days with delirium				Percentage of days with delirium	
		**n**	**Mean (SD)**	***P* value[a]**	**Total**	**Mean (SD)**	**MD (95% CI)**	***P* value[a]**	**Mean (SD)**	***P* value[a]**
Total sample	Intervention	731	11.1 (8.1)	.01	523	0.7 (2.3)	0.3 (0.1 to 0.6)	.03	5.3 (14.8)	.03
	Control	726	11.4 (7.7)		699	1.0 (2.6)			6.9 (16.7)	
Cardiac surgery	Intervention	272	10.7 (9.7)	.046	352	1.3 (3.0)	0.2 (-0.3 to 0.7)	.42	9.3 (18.4)	.42
	Control	256	11.2 (8.2)		389	1.5 (3.2)			11.0 (20.4)	
Non-cardiac surgery	Intervention	459	11.3 (6.9)	.12	171	0.4 (1.6)	0.3 (0.1 to 0.5)	.006	3.0 (11.7)	.007
	Control	470	11.6 (7.5)		310	0.7 (2.1)			4.7 (13.9)	

MD, mean difference.
[a]Mann-Whitney U test

© 2021 Deeken F et al. *JAMA Surgery*.
