# Supplementary material for: Outcomes of a Delirium Prevention Program in Older Persons After Elective Surgery: A Stepped-Wedge Cluster Randomized Clinical Trial
Source: JAMA Surg. 2021 Dec 15;157(2):e216370. doi: 10.1001/jamasurg.2021.6370 (PMC8674802; doi:10.1001/jamasurg.2021.6370)
Supplement: Supplement 3. — The PAWEL Study Group. [file jamasurg-e216370-s003.pdf]

Supplemental Online Content: Nonauthor Collaborators

*Indicates required information. Only first name, last name, and suffix will appear in PubMed.

*Group Name(s): The PAWEL Study Group							
***First Name and Middle Initial(s)**	***Last Name**	***Suffix (eg, Jr, III)**	Academic Degrees	Institution	Location (city, state/province, country)	Role or Contribution, eg, chair, principal investigator	Group (if more than 1 Group listed in the byline) and/or Subgroup (eg, Steering Committee)
Cindy	Boden		MSc	Psychotherapy at the University Hospital of Tübingen	Tübingen		Executive Committee
Stefanie	Joos		MD	University Hospital of Tübingen	Tübingen		Executive Committee
Felix	Kentischer		MScN	University of Freiburg	Freiburg		Executive Committee
Eva F.	Mennig		MSc	Klinikum Stuttgart	Stuttgart		Executive Committee
Sören	Wagner		MD	Klinikum Stuttgart	Stuttgart		Executive Committee
Jürgen	Wasem		PhD	University of Duisburg-Essen	Essen		Executive Committee
Stefan	Blumenrode		MA			Intervention Development, Training and Supervision	Intervention Development, Training and Supervision Core
Cathleen	Koch		MA			Intervention Development, Training and Supervision	Intervention Development, Training and Supervision Core
Bernd	Förstner		MSc	University of Potsdam	Potsdam	Data Management and Statistical Analysis	Data Management and Statistical Analysis Core
Andreas	Häusler		Dipl. Psych	University of Potsdam	Potsdam	Data Management and Statistical Analysis	Data Management and Statistical Analysis Core
Susanne	Schulze		MSc	University of Potsdam	Potsdam	Data Management and Statistical Analysis	Data Management and Statistical Analysis Core
Anja	Neumann		PhD	University of Duisburg-Essen	Essen	Economical Evaluation	Economical Evaluation Core
Felix	Bausenhart		MD	University Hospital of Tübingen	Tübingen		Other Co-Investigators
Manuel	Czornik		MSc	University Hospital of Tübingen	Tübingen		Other Co-Investigators
Matthias	Herrmann		MD	University Hospital of Tübingen	Tübingen		Other Co-Investigators
Andreas	Kirschniak		MD	University Hospital of Tübingen	Tübingen		Other Co-Investigators
Tobias	Krüger		MD	University Hospital of Tübingen	Tübingen		Other Co-Investigators
Marvin	Metzner		MD	University Hospital of Tübingen	Tübingen		Other Co-Investigators
Christian	Mychajliw			University Hospital of Tübingen	Tübingen		Other Co-Investigators
Petra	Renz		BN	University Hospital of Tübingen	Tübingen		Other Co-Investigators
Yuki	Schneider		MA	University Hospital of Tübingen	Tübingen		Other Co-Investigators
Andreas	Straub		MD	University Hospital of Tübingen	Tübingen		Other Co-Investigators
Heidrun	Sturm		MD	University Hospital of Tübingen	Tübingen		Other Co-Investigators
Katrin	Markgräfe-Weisser		MD	Klinikum Stuttgart	Stuttgart		Other Co-Investigators
Lorenz	Sutter		MSc	Klinikum Stuttgart	Stuttgart		Other Co-Investigators
Sarah	Weller		MSc	Klinikum Stuttgart	Stuttgart		Other Co-Investigators
Kathrin	Wunder		MD	Klinikum Stuttgart	Stuttgart		Other Co-Investigators
Fatma	Ashkanani		MD	Ulm University	Ulm		Other Co-Investigators
Dhayana	Dallmeier		MD PhD	Ulm University	Ulm		Other Co-Investigators
Clara	Dettlinger		MSc	Ulm University	Ulm		Other Co-Investigators
Jill	Holbrook		MD	Ulm University	Ulm		Other Co-Investigators
Anita	Junginger			Ulm University	Ulm		Other Co-Investigators
Helene	Maucher		RN	Ulm University	Ulm		Other Co-Investigators
Cornelia	Ribeill		MD	Ulm University	Ulm		Other Co-Investigators
Annika	Rösch		BA	Ulm University	Ulm		Other Co-Investigators
Marius	Sabau		MD	Ulm University	Ulm		Other Co-Investigators
Karl	Träger		MD	Ulm University	Ulm		Other Co-Investigators
Cynthia	Vazquez			Ulm University	Ulm		Other Co-Investigators
Ingeborg	Cuvelier		MD	KA	Karlsruhe		Other Co-Investigators
Natalie	Dudkiewicz		RN	KA	Karlsruhe		Other Co-Investigators
Janine	Peiter		RN	KA	Karlsruhe		Other Co-Investigators
Zvjezdana	Peric		SN	KA	Karlsruhe		Other Co-Investigators
Pavel	Nikolov		cand. Med	KA	Karlsruhe		Other Co-Investigators
Samina	Shah		MD	KA	Karlsruhe		Other Co-Investigators

Supplemental Online Content: Nonauthor Collaborators

*Indicates required information. Only first name, last name, and suffix will appear in PubMed.

***First Name and Middle Initial(s)**	***Last Name**	***Suffix (eg, Jr, III)**	Academic Degrees	Institution	Location (city, state/province, country)	Role or Contribution, eg, chair, principal investigator	Group (if more than 1 Group listed in the byline) and/or Subgroup (eg, Steering Committee)
Nina	Stober		MD	KA	Karlsruhe		Other Co-Investigators
Elke	Wächter		MD	KA	Karlsruhe		Other Co-Investigators
Hermann	Zöllner-Kojnov		MD	KA	Karlsruhe		Other Co-Investigators
Bernhard	Heimbach		MD	University of Freiburg	Freiburg		Other Co-Investigators
Jonas	Hoch		MA	University of Freiburg	Freiburg		Other Co-Investigators
Markus	Hören		MD	University of Freiburg	Freiburg		Other Co-Investigators
Nicole	Zimmermann		MA	University of Freiburg	Freiburg		Other Co-Investigators
Alfred	Königsrainer		MD	University Hospital of Tübingen, Visceral and General Surgery	Tübingen		Head of Department
Peter	Rosenberger		MD	University Hospital of Tübingen, Anesthesiology and Intensive Care	Tübingen		Head of Department
Christian	Schlensak		MD	University Hospital of Tübingen, Heart and Thorax Surgery	Tübingen		Head of Department
Nikolaus	Wülker		MD	University Hospital of Tübingen, Orthopedics	Tübingen		Head of Department
Thomas	Hupp		MD	Klinikum Stuttgart, Vascular Surgery	Stuttgart		Head of Department
Christian	Knop		MD	Klinikum Stuttgart, Trauma Surgery and Orthopedics	Stuttgart		Head of Department
Jörg	Köninger		MD	Klinikum Stuttgart, General Surgery	Stuttgart		Head of Department
Andreas	Walther		MD	Klinikum Stuttgart, Anaesthesiology and Intensive Care	Stuttgart		Head of Department
Andreas	Liebold		MD	Ulm University, Cardiothoracic and Vascular Surgery	Ulm		Head of Department
Heiko	Reichel		MD	Ulm University, Orthopedics	Ulm		Head of Department
Stephan	Kirschner			KA, Orthopedics, ViDia Christian Clinics	Karlsruhe		Head of Department
Uwe	Mehlhorn			KA, Helios Cardiac Surgery	Karlsruhe		Head of Department
Hagen	Schmal		MD	University of Freiburg, Trauma Surgery and Orthopedics	Freiburg		Head of Department
Stefan	Fichtner-Feigl		MD	University of Freiburg, General and Visceral Surgery	Freiburg		Head of Department
