# Supplementary material for: Outcomes of a Delirium Prevention Program in Older Persons After Elective Surgery: A Stepped-Wedge Cluster Randomized Clinical Trial
Source: JAMA Surg. 2021 Dec 15;157(2):e216370. doi: 10.1001/jamasurg.2021.6370 (PMC8674802; doi:10.1001/jamasurg.2021.6370)
Supplement: Supplement 4. — Data sharing statement. [file jamasurg-e216370-s004.pdf]

Data Sharing Statement

Deeken. Outcomes of a Delirium Prevention Program in Older Persons After Elective Surgery. *JAMA Surg*. Published December 15, 2021. doi:10.1001/jamasurg.2021.6370

Data
Data available: Yes
Data types: Deidentified participant data
How to access data: c.thomas@klinikum-stuttgart.de
When available: With publication

Supporting Documents
Document types: Statistical/analytic code, Informed consent form
How to access documents: c.thomas@klinikum-stuttgart.de
When available: With publication

Additional Information
Who can access the data: Upon reasonable request, an anonymised data set could be made available depending on the local data protection office of consent.
Types of analyses: For a specified purpose.
Mechanisms of data availability: With investigator support after approval of a proposal.
